# Localized Dimerization and Nucleoid Binding Drive Gradient Formation by the Bacterial Cell Division Inhibitor MipZ

**DOI:** 10.1016/j.molcel.2012.03.004

**Published:** 2012-05-11

**Authors:** Daniela Kiekebusch, Katharine A. Michie, Lars-Oliver Essen, Jan Löwe, Martin Thanbichler

**Affiliations:** 1 Max Planck Institute for Terrestrial Microbiology, 35043 Marburg, Germany; 2 Laboratory for Microbiology, Department of Biology, Philipps University, 35043 Marburg, Germany; 3 LOEWE Center for Synthetic Microbiology, 35043 Marburg, Germany; 4 MRC Laboratory of Molecular Biology, Cambridge CB2 OQH, UK; 5 Structural Biochemistry, Department of Chemistry, Philipps University, 35032 Marburg, Germany

## Abstract

Protein gradients play a central role in the spatial organization of cells, but the mechanisms of their formation are incompletely understood. This study analyzes the determinants responsible for establishing bipolar gradients of the ATPase MipZ, a key regulator of division site placement in *Caulobacter crescentus*. We have solved the crystal structure of MipZ in different nucleotide states, dissected its ATPase cycle, and investigated its interaction with FtsZ, ParB, and the nucleoid. Our results suggest that the polar ParB complexes locally stimulate the formation of ATP-bound MipZ dimers, which are then retained near the cell poles through association with chromosomal DNA. Due to their intrinsic ATPase activity, dimers eventually dissociate into freely diffusible monomers that undergo spontaneous nucleotide exchange and are recaptured by ParB. These findings clarify the molecular function of a conserved gradient-forming system and reveal mechanistic principles that might be commonly used to sustain protein gradients within cells.

## Graphical abstract

**Graphical Abstract:**

**Figure:**
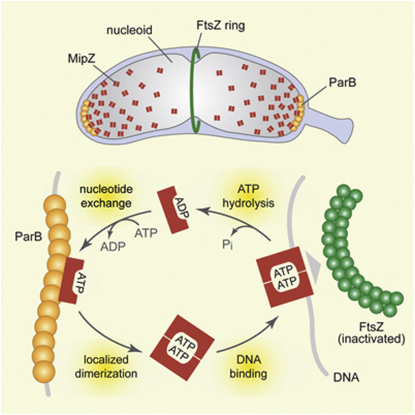

**Highlights:**

► ParB mediates ATP-dependent dimerization of MipZ at the cell poles ► MipZ dimers bind the polar regions of the nucleoid, thus directing FtsZ to midcell ► Dimers disassemble upon spontaneous ATP hydrolysis, releasing ADP-bound monomers ► Monomers undergo spontaneous nucleotide exchange and reassociate with ParB

## Introduction

Protein gradients play a central role in the regulation of biological processes. During metazoan tissue differentiation, morphogens are released from a defined group of cells and then spread into the surrounding areas. The resulting spatial gradients, shaped by a combination of diffusion and protein degradation, then lead to the differential expression of target genes in adjacent cells and thereby specify cell fate in a position-dependent manner (Wartlick et al., 2009). Similarly, intracellular protein and protein phosphorylation gradients are critical for the internal organization of both eukaryotic and prokaryotic cells, controlling the localization and activity of key factors during development, chromosome segregation, and cell division (Chen et al., 2011; Driever and Nüsslein-Volhard, 1988; Fuller et al., 2008; Martin and Berthelot-Grosjean, 2009; Moseley et al., 2009). However, although gradient-forming systems have emerged as a widespread phenomenon, the molecular mechanisms governing their formation are still incompletely understood. An excellent model to study the underlying principles is the regulation of division site placement in the dimorphic bacterium *Caulobacter crescentus*, mediated through a bipolar gradient of the cell division inhibitor MipZ (Thanbichler and Shapiro, 2006).

MipZ lies at the heart of a checkpoint that coordinates assembly of the divisome at midcell with the onset of DNA replication. Upon entry of *C. crescentus* into S phase, the two copies of its chromosomal origin region are actively segregated and sequestered to opposite cell poles (Ptacin et al., 2010; Scholefield et al., 2011). Each sister origin region carries a cluster of conserved binding sites (*parS*) that are recognized by the chromosome partitioning protein ParB (Mohl and Gober, 1997). MipZ dynamically interacts with the polar ParB-*parS* complexes, forming a subcellular gradient with concentration maxima at the tips of the cell and a minimum at the cell center (Thanbichler and Shapiro, 2006). It inhibits polymerization of the tubulin homolog FtsZ into the so-called Z ring, a structure that forms the basis of the bacterial divison apparatus (Erickson et al., 2010). Its gradient-like distribution thus prevents divisome formation in the polar regions of the cell, effectively restricting cytokinesis to the midcell region (Thanbichler and Shapiro, 2006). Due to the high diffusion rates of cytoplasmic proteins (∼8 μm^2^ s^−1^) and the small dimensions of the *C. crescentus* cell, local differences in protein levels are normally eliminated within seconds (Elowitz et al., 1999). Establishment of the MipZ gradient is therefore likely to rely on a so far uncharacterized, active process that accumulates MipZ near the cell poles.

MipZ belongs to the Mrp/MinD family of P loop ATPases, a heterogeneous group of proteins that act as nucleotide-dependent molecular switches (Leipe et al., 2002). Despite common structural and functional properties, the various family members have evolved markedly different biological functions. Of note, several of them play critical roles in the spatial regulation of bacterial cells, including the cell division regulator MinD and ParA-like chromosome partitioning proteins (Michie and Löwe, 2006). The prototypic MinD protein from *Escherichia coli* is part of an autonomous oscillatory system involved in division site placement (de Boer et al., 1989; Lutkenhaus, 2007). It forms a complex with the cell division inhibitor MinC and self-assembles on the cytoplasmic membrane in an ATP-dependent manner (Lackner et al., 2003), producing cap-like structures that line the polar regions of the cell. Driven by the ATPase-activating protein MinE, these polymers undergo rapid cycles of assembly and disassembly. Integrated over time, this process leads to a subcellular gradient in the concentration of MinCD that limits assembly of the Z ring to the cell center (Raskin and de Boer, 1999). ParA-type DNA partitioning proteins, on the other hand, are part of a molecular machinery mediating the segregation of low-copy number plasmids and bacterial chromosomes (Gerdes et al., 2010). The segregation process involves cooperative, ATP-dependent polymerization of ParA molecules into extended nucleoprotein filaments (Hui et al., 2010; Leonard et al., 2005; Ptacin et al., 2010). The ends of these filaments interact with kinetochore-like complexes that are formed through association of DNA-binding proteins (e.g., ParB) with defined centromeric sites (e.g., *parS*) on the target replicons. Stimulating the ATPase activity of adjacent ParA subunits (Easter and Gober, 2002; Leonard et al., 2005), the tethered complexes promote gradual disassembly of the filaments, thereby moving along by a ratchet-like retraction mechanism (Fogel and Waldor, 2006; Ptacin et al., 2010; Ringgaard et al., 2009; Schofield et al., 2010).

This study identifies MipZ as a distinct member of the Mrp/MinD-like ATPase family and reveals the molecular determinants that shape the steady-state MipZ concentration gradient. Our results suggest that gradient formation relies on the nucleotide-regulated cycling of MipZ between a monomeric and dimeric state with distinct interaction patterns and diffusion rates, combined with local stimulation of ATP-dependent dimerization at the polar ParB complexes. Dimers have nonspecific DNA-binding activity and are retained in the polar regions of the cell through association with the nucleoid. Their intrinsic ATPase activity acts as a timing mechanism that limits the lifetime of the dimeric complex, eventually generating monomers that rapidly diffuse back to the cell poles and, after spontaneous nucleotide exchange, restart the cycle. The MipZ gradient thus reflects the steady-state distribution of molecules in a highly dynamic system that is tuned by the kinetics of ATP binding and hydrolysis.

## Results

### MipZ Represents a Distinct Subfamily of Mrp/MinD-like ATPases

**Figure 1:**
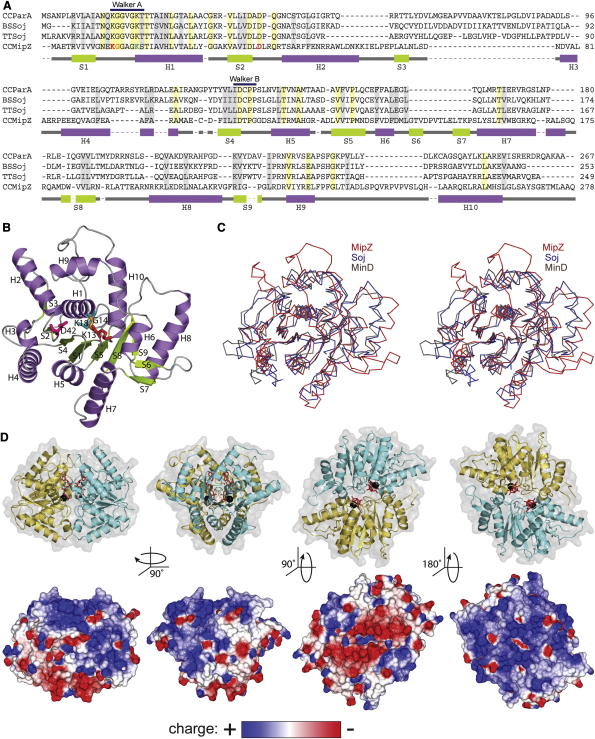
Structural Analysis of MipZ (A) Alignment of *C. crescentus* MipZ (AAK24136) with ParA/Soj homologs from *C. crescentus* (AAB51267), *B. subtilis* (CAB16134), and *T. thermophilus* (AAS81947). The sequences were aligned and then manually fitted to a structural alignment of monomeric MipZ (PDB 2XJ4) and Soj (PDB 1WCV). Invariant amino acids are shaded in yellow, similar amino acids in gray. Residues of MipZ mutated in this work are highlighted in red (K13), orange (G14), green (K18) and magenta (D42). Secondary structural elements of MipZ are indicated below the sequence in purple (α helices) and green (β sheets). (B) Crystal structure of MipZ in the nucleotide-free, monomeric state (PDB 2XJ4). The identity of secondary structural elements and the residues mutated in this work are indicated. (C) Stereo superposition of MipZ (PDB 2XJ4) with MinD from *Archaeoglobus fulgidus* (PDB 1HYQ) and Soj from *T. thermophilus* (PDB 1WCV). (D) Crystal structure of MipZ-D42A in the ATPγS-bound, dimeric state (PDB 2XJ9). Shown are the arrangement of secondary structural elements (top) and the surface electrostatic potential within a range of ± 0.3 V (bottom). See also Figure S1.

Sequence analysis identifies *C. crescentus* MipZ as a relative of MinD and ParA-like DNA partitioning proteins, with highest similarity in the N-terminal region surrounding the Walker A motif (residues 13–19 of MipZ). MipZ has no C-terminal amphipathic helix, as used for membrane attachment of MinD, and lacks the conserved DNA-binding domain characteristic of plasmid-encoded ParA proteins (Gerdes et al., 2010). Collectively, it is most similar to the ParA-type ATPases encoded by chromosomal *par* loci (Figure 1A). However, consistent with phylogenetic analyses (Figure S1A available online), its high divergence from ParA/Soj in terms of sequence (∼25% identity with *C. crescentus* ParA) and function suggests that it represents a distinct group of proteins within the Mrp/MinD ATPase family.

We crystallized MipZ in the absence of nucleotide (Table 1) and solved the structure of the apo-protein in two different space groups to 1.6 Å (PDB 2XJ4) and 1.8 Å (PDB 2XIT) resolution (Figures 1B and S1B and Table 1). The two independent structures show excellent agreement with a root-mean-square deviation (rmsd) of 0.44 Å. Apo-MipZ is monomeric, and its core adopts the same overall fold as in Soj (PDB 1WCV) and MinD (PDB 1HYQ), consisting of stacked β strands that are surrounded by α helices. Superimposition of the structures (Figure 1C) reveals that MipZ, Soj and MinD share significant similarity in the region constituting the nucleotide-binding site, with an rmsd of 2.4 Å across the 176 α-carbons forming the backbone of the conserved core. However, MipZ differs from its family relatives in the arrangement of several conserved loops. In addition, it displays a number of unique features, including a marked extension of helix 2 and a large loop between helices 9 and 10 (helices 10/11 in Soj). Notably, helices 6 and 7 of MipZ correspond to a single helix in both Soj and MinD (helix 8 in Soj). At the junction of these two helices, MipZ exhibits an unusual structural motif, consisting of a large loop that is stabilized by a short antiparallel β sheet (S6/S7). Together, these distinct characteristics are consistent with a clear evolutionary separation of MipZ from its two relatives.

### MipZ Dimerizes in a Nucleotide-Dependent Manner

Crystallization of an ATPase-deficient MipZ variant (MipZ-D42A, see below) in the presence ATPγS yielded a nucleotide-bound dimer, whose structure was solved to 2.8 Å resolution (PDB 2XJ9; Table 1). Similar to other Mrp/MinD-type ATPases (Leonard et al., 2005; Schindelin et al., 1997; Wu et al., 2011), MipZ forms a nucleotide “sandwich,” with the ATP-binding sites constituting the core of the dimer interface (Figure 1D). In addition, several residues from one subunit form key hydrogen bonds with the nucleotide associated with the other subunit, suggesting that ATP(γS) binding is critical for dimer formation. Consistent with a direct, adaptor-like role of the nucleotide in dimerization, MipZ undergoes only minor conformational changes upon transition from the apo to the ATP-bound state (Figure S1B; rmsd of 0.76 Ǻ). Most structural alterations occur in protein regions that contribute to the dimer interface, especially the P loop, the segment ranging from β sheet 5 to helix 6, and the first residues of helix 8, thereby repositioning the side chains critical for intrasubunit interaction. The MipZ dimer displays a central cleft that extends along the subunit interface down to the nucleotide-binding sites, with its bottom being lined by the phosphates of the two nucleotide moieties. The mouth of this cleft is highly negatively charged (Figure 1D, bottom, central right). The remaining surface areas of the MipZ dimer, in particular the side across the opening of the cleft (Figure 1D, bottom, right), display a high density of positive charges, thus potentially constituting a DNA-binding surface.

**Figure 2:**
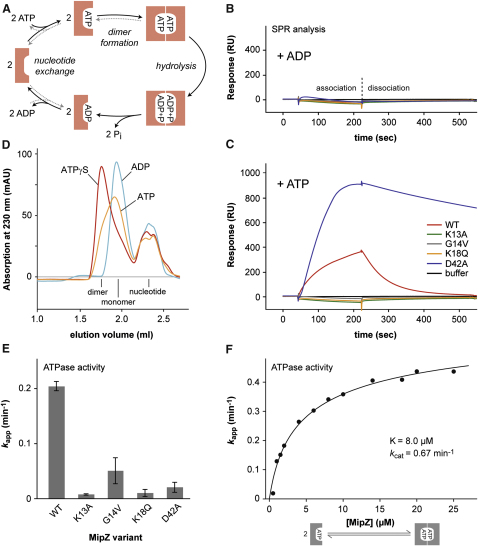
Biochemical Analysis of MipZ Dimerization (A) ATPase cycle of MipZ. See the main text for details. (B and C) SPR analysis of MipZ self-association. MipZ or its mutant derivatives (3500 RU) were immobilized on a sensor chip and tested for homotypic interaction with the respective soluble protein (0.5 μM) in the presence of ADP (B) or ATP (C). After the binding phase, the chip was washed with buffer lacking protein and nucleotides to follow the dissociation reaction. The rate constants (*k_off_*) determined for the dissociation of the MipZ and MipZ-D42A complexes are 0.83 min^−1^ and 0.067 min^−1^, respectively. (D) Nucleotide-dependent dimerization of MipZ. MipZ-D42A was incubated with ADP (blue), ATP (orange) or ATPγS (red) and subjected to size exclusion chromatography. (E) ATPase activity of MipZ and its mutant derivatives. The graph gives the average turnover rate (*k_app_*, average turnovers/min per molecule of MipZ) of the different MipZ derivatives (6 μM). Data represent the average of at least three independent experiments (±SD). Note that consistent with a critical role of the metal cofactor in dimerization and catalysis Mg^2+^ was required for ATP turnover (Figure S2H). (F) Concentration dependence of the MipZ ATPase activity. The average turnover rate of MipZ (*k_app_*) was determined at the indicated protein concentrations. Fitting of the data to a model that relates *k_app_* to the relative abundance of dimers at the indicated MipZ concentrations (see the Experimental Procedures) yielded values of 8.0 μM (±1.2 μM) for the apparent equilibrium dissociation constant (*K*) of the MipZ dimer and 0.67 min^−1^ (±0.03 min^−1^) for the turnover number (*k_cat_*) of MipZ molecules in the dimeric state. The rate of dimer formation (*k_on_* ∼ *k_cat_*/*K*) derived from these values is 1.4 × 10^3^ M^−1^ s^−1^. Data represent the mean of two independent experiments. See also Figure S2.

Previous work has shown that ATPase activity is essential for MipZ function (Thanbichler and Shapiro, 2006). To address the role of the MipZ ATPase cycle (Figure 2A) in gradient formation, we mutated four highly conserved residues in the ATP-binding domain (Figures S2A and S2B). The first exchange (K13A) targeted Lys^13^, which contacts the α- and γ-phosphates of the nucleotide bound to the adjacent subunit and might play a key role in both ATP hydrolysis and ATP-dependent dimerization. An additional substitution (G14V) replaced the highly conserved Gly^14^ in the P loop with a bulky valine residue, causing a steric clash with the γ-phosphate and the Val^14^ residue of the adjacent subunit that probably also interferes with dimer formation. The third mutation (K18Q) exchanged Lys^18^, which constitutes an integral part of the nucleotide-binding site, interacting with the β- and γ-phosphates of the nucleotide associated with the *cis* subunit. Located at the junction between the P loop and helix 1 (Figure 1A), Lys^18^ is further required for proper repositioning of the P loop upon ATP binding. Its substitution is thus predicted to reduce the affinity of MipZ for nucleotides and, in parallel, impede conformational changes required for dimerization. Finally, the highly conserved amino acid Asp^42^ was mutated to alanine (D42A). In other Mrp/MinD-type ATPases, this residue coordinates the Mg^2+^ ion and the attacking nucleophile, hence playing a pivotal role in the catalytic mechanism of ATP hydrolysis (Hayashi et al., 2001; Schindelin et al., 1997; Zhou et al., 2005).

### MipZ Dimers Form Inefficiently and Require ATP Hydrolysis for Dissociation

To dissect the ATPase cycle of MipZ, we first confirmed the ability of MipZ to self-interact in vivo using a bacterial two-hybrid assay (Figure S2C). Subsequently, we purified MipZ and its four mutant derivatives (Figure S2D) and analyzed their dimerization properties using surface plasmon resonance (SPR) analysis. Both MipZ and MipZ-D42A showed strong homotypic interaction when ATP was included in the reactions (Figures 2B and 2C). Notably, dimerization was abolished in the absence of Mg^2+^ (Figures S2F and S2G), although nucleotide binding was still observed under these conditions (Figure S3A), suggesting a role of the metal cofactor in properly orienting the critical β- and γ-phosphates of ATP. Mutations in the P loop (K13A, G14V, K18Q) of MipZ, by contrast, effectively prevented ATP-dependent complex formation. Since self-association of molecules within the analyte and ligand pools competes with the interaction monitored by SPR, the association curves only provide qualitative information. However, this issue does not apply for the dissociation curves, which directly reflect the kinetics of MipZ disassembly. Interestingly, the reduced ATPase activity of MipZ-D42A (Thanbichler and Shapiro, 2006) correlated with a slower release of the protein from the chip surface, suggesting that complex dissociation is dependent on ATP hydrolysis. In accordance with this idea, a similar decrease in the dissociation rate was observed for wild-type MipZ when incubated with slowly hydrolyzable ATPγS, while dissociation was virtually undetectable for ATPγS-bound MipZ-D42A (Figure S2E).

Further support for a close connection between the nucleotide and oligomeric state of MipZ came from gel filtration analysis (Figure 2D). Whereas MipZ-D42A behaved as a monomer in the presence of ADP, addition of ATP resulted in a partial shift to a smaller retention volume, indicative of a monomer-dimer equilibrium. When further stabilizing the complex by preincubation with ATPγS, most of the protein eluted at the smaller retention volume, verifying that MipZ in fact dimerizes in a nucleotide-dependent manner. It was not possible to detect self-association of wild-type MipZ under these conditions, probably because its higher hydrolytic potential favored complex dissociation during the experiment (data not shown).

Our results indicate a key role for nucleotide hydrolysis in dimer dissociation. To investigate whether hydrolytic activity is actually limited to the dimeric complex, we compared the ATP turnover rates of MipZ and its mutant derivatives at saturating nucleotide concentrations (Figure 2E). Substitutions in the P loop that lock MipZ in the monomeric state (Figure 2C) indeed caused a severe reduction in ATPase activity (Figure 2E), even though they still allowed for nucleotide binding (as detailed below; Table 2).

We then used the fact that only dimers possess a functional catalytic site to quantify the stability and hydrolytic activity of the dimeric complex by means of titration analysis (Figure 2F). According to the law of mass action, MipZ exists as a mixture of monomers (assumed to be largely in the ATP-bound state) and dimers, the equilibrium ratio of which depends on the total concentration of MipZ molecules in the reaction. We found the hydrolytic potential of MipZ to be drastically reduced in dilute solutions, that is conditions discouraging dimer formation. Its average turnover rate (*k_app_*) increased with rising protein concentrations and finally approached a plateau that likely reflects a situation in which all MipZ molecules have transitioned to the active, dimeric state. The data showed perfect agreement with a model correlating *k_app_* to the predicted abundance of dimers in the different conditions analyzed, with a turnover number (*k_cat_*) of 0.67 min^−1^ for each dimer subunit and an apparent equilibrium dissociation constant (*K*) of 8.0 μM.

The *k_cat_* value closely matched the dissociation rate constant (*k_off_*) determined for the MipZ dimer (compare Figure 2C). This finding adds to the conclusion that the dimeric complex forms in an ATP-dependent manner and strictly requires nucleotide hydrolysis for dissociation. However, previous work has shown that MipZ only accumulates to ∼1,000 molecules per cell (Thanbichler and Shapiro, 2006), corresponding to an average concentration of 3.5 μM. Considering the relatively high equilibrium dissociation constant, dimer formation might thus preferentially occur in the highly populated regions near the cell poles, while it is probably disfavored in pole-distal regions containing lower MipZ levels.

### MipZ Monomers Can Undergo Spontaneous Nucleotide Exchange

Once released from the dimeric complex, MipZ monomers must undergo nucleotide exchange to enter the next reaction cycle (Figure 2A). To clarify the requirements for the exchange reaction, we studied the nucleotide binding kinetics of MipZ by monitoring its interaction with methylanthraniloyl (mant)-labeled ADP and ATP analogs (Figure S3). Since the mant group blocks dimer formation, our measurements specifically focused on the initial nucleotide-binding step without interference from subsequent reactions. Analysis of the kinetic data (Table 2) showed that MipZ had comparable, and rather low, affinities for mant-ADP and mant-ATP (*K_D_* ∼ 30 μM). As expected, the K13A and D42A variants displayed similar binding characteristics as the wild-type protein, while the nucleotide binding affinity of MipZ-K18Q was strongly reduced. Surprisingly, however, the G14V mutation significantly increased the affinity of MipZ for nucleotides, probably by affecting the conformation of the P loop.

The nucleotide dissociation rate constants of MipZ closely matched those observed for small P loop GTPases in the presence of a cognate exchange factor (Klebe et al., 1995), with an average lifetime (τ = 1/*k_off_*) for the MipZ⋅mant-ADP complex of about 0.3 s (Table 2). This finding suggests that MipZ can undergo spontaneous nucleotide exchange once the two subunits have been released from the post-hydrolysis complex. After nucleotide dissociation, both ADP and ATP compete for binding to the ligand-free subunit. However, despite their similar association rate constants, the high cellular ATP/ADP ratio will strongly favor formation of the MipZ⋅ATP complex in vivo, thus rendering the exchange reaction quasi-irreversible. Collectively, our results show that, next to dimer formation, hydrolysis-driven dissociation of the sandwich dimer (τ ∼ 1/*k_cat_* = 90 s) represents a rate-limiting step in the ATPase cycle of MipZ. Spontaneous nucleotide exchange, by contrast, occurs comparably fast, thereby quantitatively converting monomers back to the dimerization-competent, ATP-bound state.

### Productive Interaction with FtsZ Requires MipZ Dimerization

**Figure 3:**
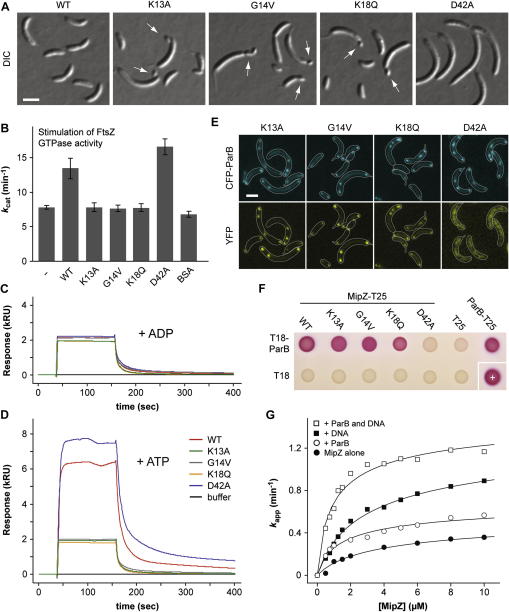
Interaction of MipZ with FtsZ and ParB (A) Effect of different mutations on the activity of MipZ. Strains MT177 (WT), DK2 (K13A), MT178 (G14V), MT179 (K18Q), and MT197 (D42A) were cultivated for 8 hr in conditions that prevent expression of wild-type *mipZ* while inducing expression of the indicated *mipZ*-*yfp* allele (the scale bar represents 2 μm). (B) Effect of MipZ and its mutant derivatives on the GTPase activity of FtsZ. Shown are the turnover numbers (*k_cat_*) of FtsZ in the absence of additional proteins (–) or in the presence of different MipZ variants or BSA. Data represent the average of at least three independent experiments (±SD). (C and D) SPR analysis of the interaction between MipZ and ParB. ParB (3800 RU) was immobilized on a sensor chip and probed with the indicated MipZ proteins (6 μM) in the presence of ADP (C) or ATP (D). (E) Colocalization of mutant MipZ variants with ParB. Strains DK3 (K13A), DK5 (G14V), DK6 (K18Q), and DK7 (D42A) were induced for 8 hr to synthesize CFP-ParB and the indicated MipZ-YFP variants in place of wild-type MipZ (the scale bar represents 2 μm). Note that depletion of wild-type MipZ did not affect the interaction of ParB with the chromosomal origin regions (data not shown). (F) Two-hybrid analysis of the interaction between MipZ and ParB. *E. coli* BTH101 was transformed with pairs of plasmids encoding the indicated derivatives of the T18 and T25 fragments of adenylate cyclase, respectively. Interaction is evidenced by the formation of red colonies. Positive control (+): T25-zip:T18-zip. (G) Effect of ParB on the ATPase activity of MipZ. The average turnover number (*k_app_*) of MipZ was determined over a range of different MipZ concentrations. When appropriate, the reactions were supplemented with ParB and/or *parS*-containing plasmid DNA (pMT428; 3.6 kb). The data were approximated by the model used in Figure 2F, yielding the following *K* and *k_cat_* values: MipZ alone (7.1 μM and 0.61 min^−1^), + ParB (2.6 μM and 0.77 min^−1^), + DNA (6.0 μM and 1.5 min^−1^), + ParB and DNA (1.6 μM and 1.6 min^−1^). See also Figure S4.

To investigate the role of nucleotide-regulated dimerization in the function of MipZ, we analyzed cell division in strains synthesizing constitutively monomeric (K13A, G14V, K18Q) or dimeric (D42A) variants of MipZ in place of the endogenous wild-type protein (Figure 3A). Substitutions in the P loop (K13A, G14V, K18Q) consistently produced the same minicell phenotype as MipZ depletion (Thanbichler and Shapiro, 2006), suggesting that the mutant proteins have lost the ability to control FtsZ positioning. The D42A exchange, by contrast, resulted in a complete block of cytokinesis, in line with the previous finding that it generates a deregulated form of MipZ preventing FtsZ ring assembly throughout the cell (Thanbichler and Shapiro, 2006). MipZ was shown to inhibit the polymerization of FtsZ in an ATP-dependent manner by stimulating the FtsZ GTPase activity (Thanbichler and Shapiro, 2006). We found that this stimulatory effect was exclusively observed for the dimerization-competent forms of MipZ (WT and D42A) but not for its monomeric derivatives (Figures 3B and S4A). Consistent with the in vivo data, self-association of MipZ thus appears to be critical for its productive interaction with FtsZ, implying that division site placement is effectively controlled by the subcellular distribution of MipZ dimers.

### ParB Acts as a Polar Sink for MipZ Monomers

MipZ is recruited to the polar regions of the cell through direct interaction with the origin-bound ParB complex (Thanbichler and Shapiro, 2006). To clarify the precise contribution of ParB to gradient formation, we investigated the role of nucleotide binding and dimerization in the recruitment process using SPR analysis. Consistent with previous evidence for a direct interaction between ParB and apo-MipZ (Thanbichler and Shapiro, 2006), all MipZ variants associated with a ParB-coupled sensor surface in the nucleotide-free (data not shown) and ADP-bound (Figure 3C) state. Upon addition of ATP, the dimerization-competent (WT and D42A) proteins showed an increased response, possibly reflecting the binding of dimers and/or the formation of more stable complexes (Figure 3D). Due to the concentration dependence of MipZ self-association (Figure 2F), it was not possible to determine the precise binding affinities by titration analysis. However, qualitatively, these results demonstrate that MipZ can interact with ParB independent of its nucleotide and oligomerization state, consistent with the lack of major nucleotide-induced rearrangements in its surface structure.

To validate the SPR data, we analyzed the different MipZ variants for their ability to associate with ParB in vivo. Cells depleted of the endogenous MipZ protein exhibit severe division defects that often entail the accumulation of supernumerary chromosomes, as indicated by the formation of multiple ParB complexes (Thanbichler and Shapiro, 2006). Consistent with the SPR results, the monomeric proteins (K13A, G14V, and K18Q) assembled into foci that colocalized with ParB in this depletion background (Figure 3E). The D42A variant, by contrast, was distributed throughout the cell, forming a patchy pattern that was not correlated to the subcellular position of ParB clusters. In agreement with the localization data, bacterial two-hybrid analysis revealed that only wild-type MipZ and its monomeric variants were able to bind ParB, whereas no positive signal was detected for the D42A protein (Figure 3F). Collectively, these results suggest the existence of a competing interaction (as detailed below) that prevents association of MipZ dimers with ParB in vivo, thereby effectively restricting access to the origin-bound ParB clusters to monomeric MipZ.

Previous work has shown that, in the presence of *parS*-containing DNA, ParB stimulates the ATPase activity of ParA/Soj up to 60-fold (Leonard et al., 2005; Scholefield et al., 2011), probably by accelerating the rate of nucleotide hydrolysis. Since MipZ does not interact with ParB after transition to the hydrolysis-competent, dimeric state under in vivo conditions, its catalytic cycle is unlikely to rely on a similar mechanism. To clarify this issue, we reanalyzed the concentration dependence of MipZ's ATPase activity (compare Figure 2F) in the presence of both ParB and/or a plasmid that carried a chromosomal fragment containing all predicted *C. crescentus parS* sites (Figure 3G). Addition of DNA led to a slight (∼2-fold) increase in the catalytic activity of the dimeric complex (*k_cat_*), although it did not change the monomer-dimer equilibrium (*K*). Consequently, the MipZ dimer might be able to interact with DNA independently of ParB (as detailed below), leading to conformational adjustments that enhance its hydrolytic potential. Upon addition of ParB, by contrast, the catalytic activity of the dimer did not change. However, the MipZ concentrations required to reach the maximum turnover rate were significantly lower, as reflected by a marked (∼4-fold) decrease in the apparent equilibrium dissociation constant of the dimeric complex. Notably, the stimulatory effect of ParB was not dependent on *parS* but also observed in the absence of DNA (Figure 3G) and in the presence of a plasmid lacking *parS* sites (data not shown). Collectively, these findings argue against a role of ParB as an ATPase-activating factor. Rather, they suggest that ParB might have a positive influence on dimer formation, thereby facilitating transition of MipZ to the hydrolysis-competent state.

### The MipZ Dimer Binds to DNA in a Nonspecific and Noncooperative Manner

**Figure 4:**
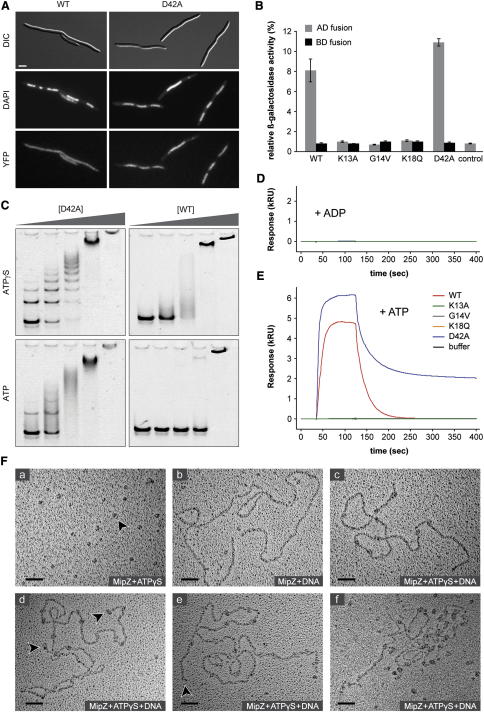
ATP-Dependent Interaction of MipZ with DNA (A) Interaction of MipZ with the *E. coli* nucleoid. Cells of *E. coli* TOP10 producing the indicated MipZ-YFP variants were treated with cephalexin and chloramphenicol to induce filamentous growth and nucleoid condensation, respectively. DNA was stained with 4′,6-diamidino-2-phenylindole (DAPI) (the scale bar represents 3 μm). Note that the fusion proteins accumulated to the same level (data not shown). (B) Detection of the DNA-binding activity of MipZ by yeast one-hybrid analysis. Yeast strains producing fusions of the indicated MipZ variants to the GAL4 transactivation domain were assayed for the expression of a β-galactosidase reporter gene. Activities were normalized to the level determined for the GAL4 positive control. Data represent the average of three independent measurements (±SD). (C) Probing of the interaction between MipZ and DNA by EMSA. A non-specific DNA fragment was incubated with 12- to 300-fold molar excess of wild-type MipZ or MipZ-D42A in the presence of ATP or ATPγS and subjected to gel electrophoresis. (D and E) SPR analysis of the interaction between MipZ and DNA. A 26-bp long, double-stranded oligonucleotide (ran2) was immobilized on the sensor surface (500 RU) and probed with the indicated MipZ variants (6 μM) in the presence of ADP (D) or ATP (E). Very similar results were obtained for oligonucleotides *parS*_WT_, *parS*_mt_ and ran1 (data not shown). (F) Visualization of MipZ-DNA complexes. MipZ-D42A was incubated with ATPγS (a), linearized phage ΦX174 DNA (b), or both ATPγS and DNA (c–e). Complexes were detected by rotary shadowing electron microscopy (scale bars represent 50 nm). Arrowheads highlight putative MipZ-D42A dimers. Panel (f) shows the same experiment as (c)–(e), performed at a 32-fold higher concentration of MipZ-D42A. See also Figure S5.

Our localization studies revealed that MipZ-D42A had a somewhat patchy subcellular distribution, suggesting that the dimeric complex was not freely diffusible. Prompted by the nonspecific DNA-binding activity of ParA-like ATPases (Castaing et al., 2008; Hui et al., 2010; Leonard et al., 2005), we therefore assessed the ability of MipZ to associate with the nucleoid. For this purpose, YFP fusions of the different MipZ variants were produced heterologously in *Escherichia coli*, a bacterium that lacks both MipZ and a ParAB-based chromosome partitioning system. Microscopic analysis revealed that the K13A, G14V and K18Q derivatives (data not shown) as well as the wild-type protein (Figure 4A) were evenly dispersed throughout the cells. The D42A variant, by contrast, consistently colocalized with the nucleoids, suggesting that MipZ is indeed capable of interacting with DNA when in its ATP-bound, dimeric state. The diffuse distribution of the wild-type protein can be explained by its elevated ATPase activity and, possibly, the absence of ParB, which might raise the concentration of untethered monomers and thus mask the nucleoid-associated signal.

Further evidence for a DNA-binding activity of MipZ came from yeast one-hybrid analyses (Figure 4B). Both wild-type MipZ and its D42A variant stimulated transcription of a β-galactosidase reporter gene when fused to the GAL4 transactivation domain, whereas the constitutively monomeric variants did not show any effect (Figure 4B). These results support the notion that dimerization triggers non-specific interaction of MipZ with DNA, thereby promoting delivery of the attached transactivation domain to the GAL4-dependent β-galactosidase promoter region.

To exclude artifacts potentially associated with in vivo analyses, we investigated the DNA-binding properties of MipZ in a purified system using electrophoretic mobility shift assays (EMSA). In the presence of slowly hydrolyzable ATPγS, both wild-type MipZ and MipZ-D42A showed a strong and concentration-dependent interaction with a nonspecific 282 bp dsDNA fragment (Figure 4C). However, whereas reactions containing MipZ-D42A gave rise to a number of discrete nucleoprotein complexes, wild-type MipZ produced a continuous smear, suggesting that it dissociated from its target during electrophoresis. The efficiency of complex formation was generally lower when the assay was performed with ATP instead of ATPγS, reaching almost background levels in case of wild-type MipZ. The binding activity thus dropped as the potential for nucleotide hydrolysis increased, consistent with the idea that only the ATP-bound, dimeric form of MipZ can stably associate with DNA.

To further clarify the requirements for DNA binding, we monitored the interaction of MipZ and its mutant derivatives with short, double-stranded oligonucleotides using SPR analysis. The target molecules comprised either a single *parS* motif (*parS*_WT_), a mutated version of *parS* (*parS*_mt_), or random sequences (ran1 and ran2). While interaction was undetectable in the absence of nucleotides (data not shown) or in the presence of ADP (Figure 4D), the dimerization-competent forms of MipZ (WT and D42A) produced strong binding signals for all oligonucleotides in ATP-containing buffer (Figure 4E). MipZ required Mg^2+^ for DNA binding (Figure S5A) and also interacted with single-stranded DNA, but not with RNA or polyphosphate (Figure S5B). Notably, the protein was released from its target in two distinct phases, which might reflect dissociation from secondary (e.g., DNA ends) and full binding sites, respectively, as indicated by the absence of the fast component at lower protein concentrations (Figures S5C and S5D). For the D42A variant, the second, slower phase was significantly longer, consistent with the increased lifetime of its dimeric state (compare Figure 2C). Although the concentration dependence of MipZ dimerization (Figure 2F) again precluded quantitative measurements, these results collectively suggest that MipZ dimers bind DNA in a nonspecific manner.

ParA-like plasmid and chromosome partitioning ATPases interact cooperatively upon DNA binding, forming extensive nucleoprotein filaments (Castaing et al., 2008; Leonard et al., 2005). To determine the binding mode of MipZ, we visualized complexes of MipZ-D42A and nonspecific ΦX174 DNA by rotary-shadowing electron microscopy (Figure 4F). When incubated with ATPγS in the absence of DNA, the protein appeared as small, regular “blobs,” consistent with the formation of dimers. Samples of protein and DNA that lacked nucleotide displayed strings of nucleic acid and dot-like particles probably representing MipZ monomers, without any sign of interaction between the two molecule species. Upon addition of ATPγS, by contrast, densities corresponding to putative dimeric complexes were associated with the DNA. Independent of the protein concentration used, these structures were well separated and spaced at apparently random intervals, suggesting that DNA binding occurs in a noncooperative fashion.

Interestingly, competition assays revealed that DNA and ParB associate with MipZ dimers in a mutually exclusive fashion, suggesting overlapping binding sites (Figures S4B, S5C, and S5D). The high intracellular concentration of chromosomal DNA is thus likely to shift the equilibrium toward the DNA-bound state in vivo, thereby quantitatively immobilizing MipZ dimers on the nucleoid and preventing their sequestration to the polar ParB complexes. This finding might explain why MipZ dimers fail to associate with ParB in the cellular context, although interaction is clearly observed in a purified system.

### MipZ Exhibits Nucleotide-Regulated Diffusion Kinetics

**Figure 5:**
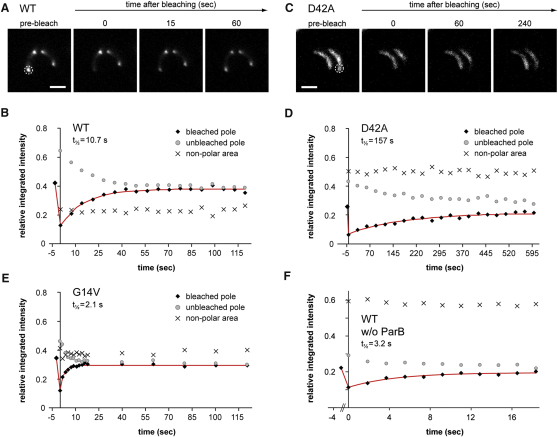
FRAP Analysis of MipZ Diffusion (A and C) FRAP experiments on cells producing a wild-type MipZ-YFP fusion (A) or a MipZ-D42A fusion (C). Cells of strain MT177 (WT) or MT197 (D42A) were shifted from M2G_N_ to M2G medium containing 0.3% xylose and cultivated for another 4 hr to deplete wild-type MipZ and synthesize the indicated fusion protein instead. Single polar MipZ-YFP foci were photo-bleached (white circles), and recovery was followed by fluorescence microscopy (scale bars represent 2 μm). (B, D, and E) Quantification of the recovery rate in cells producing YFP fusions to wild-type MipZ (MT177) (B), MipZ-D42A (MT197) (D), and MipZ-G14V (MT178) (E). FRAP experiments were performed as described in (A). The graphs show the average relative integrated intensities of the bleached region, an unbleached region of equal size at the opposite pole, and the remaining area of the cell plotted as a function of time. The recovery half-times (t_1/2_), as determined by fitting of the data to single-exponential functions (red lines), are given on top of the graphs. We verified that expression from an inducible promoter did not affect the dynamics of MipZ-YFP (Figure S6C). (F) Quantification of the recovery rate of MipZ-YFP in cells depleted of ParB. Strain MT148 was grown in M2G medium for 15 hr and analyzed as described in (B), (D), and (E). The culture did not contain any anuclate cells. The recovery half-time measured before depletion of ParB was 11.8 s (±1.2 s; n = 34) (data not shown). See also Figure S6.

Due to their association with chromosomal DNA, MipZ dimers should display slower diffusion rates than monomers. To test this hypothesis, we monitored the mobility of wild-type and mutant MipZ-YFP fusions using fluorescence recovery after photobleaching (FRAP) analysis. When a short laser pulse was applied to one of the cell poles in predivisional cells synthesizing an inducible wild-type fusion, polar foci were fully restored within 1 min after the bleaching event (Figure 5A). Quantitative, time-resolved measurements yielded a recovery half-time (t_1/2_) of 10.7 s (±0.9 s; n = 24) (Figure 5B). Concurrent with the reappearance of fluorescence at the bleached pole, the intensity of the unbleached focus decreased, while the total integrated fluorescence remained constant within the cell. Almost identical results were obtained in the presence of the translational inhibitor chloramphenicol (Figure S6B), indicating that the observed dynamics are based on the exchange of MipZ molecules between the two cell poles.

**Figure 6:**
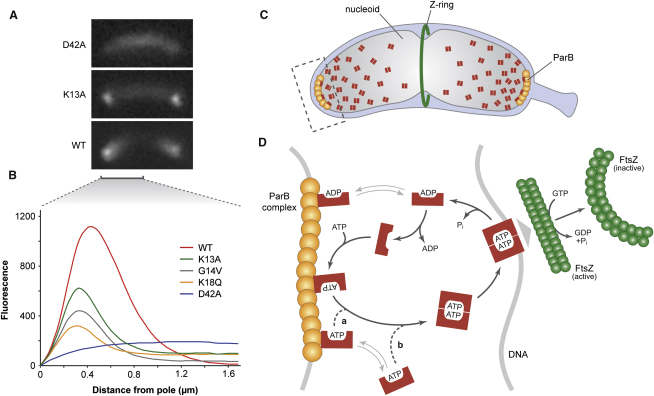
Model of MipZ Gradient Formation (A and B) Subcellular localization of MipZ variants. Strains MT177, DK2, MT178, MT179, and MT197 were depleted of wild-type MipZ and induced to synthesize the indicated MipZ-YFP variants. After 4 hr of incubation, cells were imaged and early predivisional stalked cells with wild-type morphology were selected for further analysis. Fluorescence intensity profiles were recorded for 82-115 cells per strain and averaged. Panel (A) shows representative fluorescence micrographs of cells producing wild-type MipZ-YFP (WT) or mutant variants locked in the monomeric (K13A) or dimeric (D42A) state. (C) Gradient-like distribution of MipZ dimers over the nucleoid of a *C. crescentus* predivisional cell. Dimers form in proximity of the ParB complexes and are retained in the polar regions of the cell through non-specific interaction with chromosomal DNA. The hatched box indicates the cellular region magnified in (D). (D) Nucleotide-regulated cycling of MipZ between the polar ParB complex and chromosomal DNA. ParB could promote MipZ dimerization either (a) directly by acting as a catalyst (M. Schmidt and P. Lenz, personal communication) or (b) indirectly by increasing the local concentration of MipZ at the cell poles.

Importantly, the recovery rate measured was in reasonable agreement with the rate constant obtained for the dissociation of MipZ from DNA in vitro (*k_off_* = 1.5 min^−1^; half-time t_1/2_ = 28 s; Figure S5C). The majority of MipZ-YFP molecules might thus exist as dimers that are sequestered to the pole-proximal regions of the nucleoid, with recovery depending on a small pool of mobile monomers generated by spontaneous nucleotide hydrolysis. In support of this hypothesis, the diffusely localized (Figures 6A and 6B), hydrolysis-impaired D42A variant indeed displayed very slow recovery kinetics (t_1/2_ = 157 ± 28 s; n = 19; Figures 5C and 5D), indicating that transition to the DNA-binding, dimeric state slows down diffusion of MipZ in vivo. The constitutively monomeric proteins (K13A, G14V, and K18Q), by contrast, were highly mobile, with a recovery half-time of 2.1 s (±0.2 s; n = 29) for the G14V variant (Figure 5E) and similar values being measured for the K13A and K18Q derivatives (Figures S6D and S6E). Notably, all monomeric species failed to form the characteristic gradient-like pattern observed for wild-type MipZ and instead showed distinct polar foci, reflecting their association with the origin-bound ParB complexes, as well as a significant background of evenly dispersed protein (Figures 6A and 6B). Nucleotide-regulated alternation of MipZ between a fast- and a slow-diffusing state thus appears to be critical for gradient formation.

The differential diffusion rates of monomers and dimers provided a means to analyze the effect of ParB on MipZ dimerization in vivo. Previous work has shown that MipZ-YFP is delocalized in cells depleted of ParB (Thanbichler and Shapiro, 2006). Using FRAP to analyze the mobility of the dispersed protein (Figure 5F), we obtained a recovery half-time of 3.2 s (±0.6 s; n = 25), indicating that the majority of MipZ molecules had transitioned to the monomeric state. Consistent with the in vitro data (Figure 3G), this finding supports a key role of ParB in the formation and proper localization of MipZ dimers.

## Discussion

The MipZ system provides a striking example of a steady-state protein concentration gradient established within the cytoplasm of a bacterial cell. Confining assembly of the Z ring to midcell, it plays a central role in the regulation of cell division in *C. crescentus* and, probably, many other alpha-proteobacteria. Our findings indicate that gradient formation relies on nucleotide-regulated alternation of MipZ between its monomeric and dimeric state, which in turn translates into dynamic changes in its interaction network and diffusion rate. The underlying mechanism is distinct from the large-scale spatial oscillations that drive the Min system in *E. coli* and also markedly deviates from the regime of localized protein synthesis and diffusion observed for steady-state morphogen gradients in metazoa.

### Mechanism of MipZ Gradient Formation

We propose that the differential mobility of monomers and dimers provides the basis for the gradient-like subcellular distribution of MipZ by facilitating retention of the dimeric division inhibitor complex in the polar regions of the cell (Figures 6C and 6D). Interaction analyses showed that MipZ monomers bind to ParB irrespective of their nucleotide state (Figures 3C–3E). Consistent with the moderate affinity of this interaction (*K_D_* ∼ 2 μM for apo-MipZ) (Thanbichler and Shapiro, 2006), the MipZ-ParB complex exchanges subunits rapidly, with turnover times of only a few seconds (Figure 5E). The polar ParB clusters thus act as sinks that collect freely diffusible MipZ molecules and release them in proximity of the poles. Importantly, the total cellular concentration of MipZ (3.5 μM) is significantly lower than the apparent equilibrium dissociation constant of the dimeric complex (8 μM), which should prevent efficient dimerization. Nevertheless, most of the MipZ molecules exist as dimers in vivo (Figure 5B), with a bias in their localization toward the polar regions of the cell. The sink function of ParB might therefore lead to a sharp increase in the local concentration of MipZ at the cell poles, which in turn entails a shift of the equilibrium toward the dimeric state. Although the MipZ dimers generated can still interact with ParB (Figure 3D), the huge excess of chromosomal binding sites causes their relocation to the nucleoid (Figure S4B). Slowed down by association with the dense matrix of chromosomal DNA (Figure 5D), most of the dimers are retained in the pole-proximal regions of the cytoplasm, while only some succeed in moving farther toward midcell. Based on this filter effect, the density of MipZ-DNA complexes decreases as a function of distance from the pole. The intrinsic ATPase activity of MipZ acts as a timing mechanism that limits the life span of the dimeric complex and ensures that MipZ molecules periodically transition to the monomeric state. Monomers are unable to interact with the nucleoid and, consequently, display high diffusional mobility (Figures 5E, S6D, and S6E), thereby rapidly returning to the polar ParB clusters. In simplified form, the MipZ gradient can thus be envisioned as the skewed distribution of MipZ dimers that assemble from a polar pool of monomeric subunits, diffuse slowly toward midcell, and then disassemble due to ATP hydrolysis. Of note, only the dimeric complex can productively interact with FtsZ, while the pool of freely diffusible monomers has no effect on Z-ring formation (Figures 3B and S4A) (Thanbichler and Shapiro, 2006). Conversely, FtsZ does not affect the ATPase activity or subcellular distribution of MipZ (data not shown), excluding the possibility that the Z ring contributes to shaping the MipZ gradient. This mechanistic framework provides the basis for future modeling studies that will help to fully unravel the dynamics of the MipZ system.

### Comparison of MipZ with Related Mrp/MinD Family Members

Phylogenetic analysis showed that MipZ orthologs form a distinct group within the Mrp/MinD family of ATPases, with highest similarity to ParA-type DNA partitioning proteins (Figure S1A). MipZ indeed shares several key features with chromosomally encoded ParA/Soj proteins, such as the structure of its catalytic core, its ability to interact with ParB, and its nonspecific DNA-binding activity, supporting a similar evolutionary origin. However, despite their overall resemblance, the properties of MipZ and ParA/Soj have diverged significantly. MipZ displays unique structural characteristics that clearly differentiate it from its family relatives. Importantly, it lacks the two hydrophobic clefts that extend from the surface of the Soj dimer down to its catalytic sites. These openings were proposed to accommodate the N-terminal amino acids of Spo0J (ParB), among them a conserved arginine residue that might participate in nucleotide hydrolysis and thus mediate the stimulatory effect of Spo0J on the ATPase activity of Soj (Leonard et al., 2005; Scholefield et al., 2011). The distinct architecture of MipZ probably excludes this regulatory peptide from the catalytic site. Moreover, we found that MipZ dimers are stably tethered to the nucleoid (Figures 4 and 5C) and hence unable to efficiently interact with the pole-associated ParB complex (Figures 3E and 3F). Based on these findings, ParB is unlikely to be involved in the catalytic mechanism of nucleotide hydrolysis, as supported by its rather modest effect on ATP turnover in vitro (Figure 3G). Furthermore, it cannot have a critical role in dimer dissociation or nucleotide exchange, given the low affinity between ADP-bound monomers (Figures 2B and 2D) and the high rate of spontaneous ADP release (Table 2). The key function of ParB therefore appears to lie in the stimulation of dimer formation, both through concentration of MipZ at the cell poles and, potentially, a catalytic effect on its dimerization (Figures 3G and 5F). Collectively, these results reveal that MipZ and ParA/Soj show clear differences in the use of ParB and the regulation of their ATPase cycles, which directly translate into distinct interaction and localization dynamics.

Our results exemplify the intriguing diversity of Mrp/MinD-like ATPases. Based on a similar fold, the different family members have evolved widely divergent regulatory patterns and interaction networks that specify their particular biological role. Acting as nucleotide-regulated molecular switches, they show functional similarity with Ras-like GTPases, a largely eukaryote-specific group of regulators with which they indeed share a common evolutionary origin (Leipe et al., 2002). Thus, prokaryotes and eukaryotes use similar classes of proteins to regulate key processes in cell biology.

### Conserved Features of Intracellular Protein Concentration Gradients

Protein concentration gradients have emerged as a common theme in the regulation of cellular processes. A prominent recent example for this regulatory strategy is the control of cell size homeostasis in *Schizosaccharomyces pombe*, based on bipolar gradients of the protein kinase Pom1 (Martin and Berthelot-Grosjean, 2009; Moseley et al., 2009). Interestingly, despite differences in the mechanistic details, the general principles shaping the Pom1 and MipZ gradients are strikingly similar. Phosphorylated Pom1 is recruited to the tips of the cell through interaction with a polar landmark protein (Hachet et al., 2011). After local dephosphorylation, it associates with the cortical membrane and slowly spreads across the polar regions of the cell. Autophosphorylation then gradually reduces the membrane affinity of Pom1 and promotes its dissociation from the cortex. The soluble form of Pom1 diffuses rapidly within the cytoplasm and finally relocates to the cell poles. Thus, in both *S. pombe* and *C. crescentus*, gradient formation relies on a dynamic localization cycle driven by the alternation of a regulatory protein between two different states with distinct diffusion rates. These parallels suggest that common principles might apply to the establishment of intracellular protein concentration gradients in all domains of life.

## Experimental Procedures

### Strains, Plasmids, Media, and Growth Conditions

The strains and plasmids used in this study are listed in Tables S1 and S2. Details on their construction and details on the growth conditions used are given in the Supplemental Experimental Procedures.

### Light and Electron Microscopy

For light microscopy, cells were grown to midexponential phase and induced with D-xylose, myo-inositol or L-arabinose as indicated. Subsequently, they were transferred onto a 1%-agarose pad and visualized using an Axio Imager.M1 microscope (Zeiss), a Zeiss Plan Apochromat 100×/1.40 Oil DIC objective and a Cascade:1K CCD camera (Photometrics). Images were processed with MetaMorph 7.1.2 (Molecular Devices) and Adobe Photoshop CS2 (Adobe Systems). Fluorescence intensity profiles were measured using the line scan function of MetaMorph 7.2.1 (maximum value; line width: 8). FRAP analysis and rotary shadowing transmission electron microscopy were performed as described in the Supplemental Experimental Procedures.

### Protein Expression, Purification, Crystallization, and Data Collection

Hexahistidine-tagged MipZ was purified by Ni-NTA affinity chromatography and subsequent ion exchange chromatography and/or gel filtration (see the Supplemental Experimental Procedures). Native ParB and FtsZ were prepared as described previously (Thanbichler and Shapiro, 2006). Details on crystallization and data collection are given in the Supplemental Experimental Procedures.

### Enzymatic Activity Assays

β-galactosidase, MipZ, and FtsZ activity assays are detailed in the Supplemental Experimental Procedures. To analyze the concentration dependence of MipZ's ATPase activity, the data were fitted to the following equation using Origin 6.1 (OriginLab):

$$k_{app}=2k_{cat}\frac{\left[MM\right]}{\left[M_{t}\right]}=2k_{cat}\left(\frac{1}{2}+\frac{K-\sqrt{8\left[M_{t}\right]K+K^{2}}}{8\left[M_{t}\right]}\right),$$

whereby *k_app_* is the observed average turnover rate, *k_cat_* the turnover number of MipZ molecules in the dimeric complex, [MM] the dimer concentration, [M_t_] the total concentration of MipZ molecules in the reaction, and K the equilibrium constant [M]^2^/[MM]. [M] represents the concentration of MipZ monomers, which are assumed to be largely in the ATP-bound state.

### Molecular Interaction Analyses

Details on transient kinetic analysis, surface plasmon resonance analysis, size exclusion chromatography and electromobility shift assays are given in the Supplemental Experimental Procedures.

## Figures and Tables

**Table 1:** Crystallographic Data and Refinement Statistics

	MipZ (Apo-Protein)	MipZ (Apo-Protein)	MipZ:ATPγS, Mg^2+^ (Dimer)
**Crystallographic Data**			

Space group	H3	H32	P4_1_
Unit cell (Å)	a = b = 81.33, c = 124.21	a = b = 124.67, c = 239.05	a = b = 57.12, c = 164.86
λ (Å)	0.9747	0.8726	0.8726
Resolution (Å)	1.6	1.8	2.8
*I*/σ*I*^a^	11.6 (5.1)	21.0 (4.9)	8.8 (2.7)
R_m_^b^ (%)	0.090 (0.290)	0.057 (0.383)	0.079 (0.349)
Multiplicity for unique reflections	5.4 (5.5)	6.0 (5.5)	2.5 (2.5)
Completeness for unique reflections (%)	99.6 (99.6)	99.9 (100.0)	94.2 (96.5)

**Refinement Statistics**			

Model	4–273, 353 H_2_O	Chain A: 2–287; Chain B: 4–287, 420 H_2_O	Chain A: 3–148, 151–191, 193–270; Chain B: 3–175, 178–240, 243–272, 2 ATP, 59 H_2_O
R factor, R free^c^	0.126, 0.175	0.185, 0.230	0.185, 0.240
B average/bonded^d^	23.7 Å^2^, 3.0 Å^2^	30.8 Å^2^, 1.6 Å^2^	49.3 Å^2^, 7.2 Å^2^
Geometry bonds/angles^e^	0.03 Å, 2.474°	0.012 Å, 1.294°	0.010 Å, 1.530°
Ramachandran^f^	95.8, 0.0	98.0, 0.9	86.5, 0.0
PDB ID	2XJ4	2XIT	2XJ9

Highest-resolution bins are in brackets.

a Signal to noise ratio for the highest resolution shell of intensities.

b R_m_: Σ*_h_*Σ*_i_* | *_I_*(*h,i*) – *I*(*h*) | / Σ*_h_*Σ*_i_ I*(*h,i*) where *I*(*h,i*) are symmetry related intensities and *I*(*h*) is the mean intensity of the reflection with unique index *h*.

c Five percemt of reflections were randomly selected for determination of the free R factor, prior to any refinement.

d Temperature factors averaged for all atoms and rmsd of temperature factors between bonded atoms.

e Rmsds from ideal geometry for bond lengths and restraint angles.

f Percentage of residues in the “most favored region” of the Ramachandran plot and percentage of outliers.

**Table 2:** Biochemical Parameters of Nucleotide Binding

Protein	*k*_on_ (M^−1^s^−1^)	*k*_off_ (s^−1^)	K_D_ (μM)^a^
**mant-ATP**			

WT	6.8 × 10^4^	2.1	31
K13A	5.6 × 10^4^	2.1	38
G14V	15.7 × 10^4^	0.2	1.3
K18Q	6.2 × 10^4^	7.5	121
D42A	7.8 × 10^4^	4.5	58

**mant-ADP**			

WT	11.8 × 10^4^	3.1	26
K13A	8.6 × 10^4^	3.5	41
G14V	20.5 × 10^4^	0.9	4.5
K18Q	ND	ND	ND
D42A	5.5 × 10^4^	3.8	69

ND, not detectable. See also Figure S3.

a Calculated using the kinetic parameters (K_D_ = *k*_off_/*k*_on_).

## Accession Numbers

The atomic coordinates of apo-MipZ (PDB 2XJ4 and PDB 2XIT) and ATPγS-bound dimeric MipZ (PDB 2XJ9) have been deposited in the RCSB Protein Data Bank.

## Supplemental Information

Document S1. Figures S1–S6, Tables S1–S3, and Supplemental Experimental Procedures
