# Supplementary material for: Localized Dimerization and Nucleoid Binding Drive Gradient Formation by the Bacterial Cell Division Inhibitor MipZ
Source: Mol Cell. 2012 May 11;46(3):245–59. doi: 10.1016/j.molcel.2012.03.004 (PMC3355305; doi:10.1016/j.molcel.2012.03.004)
Supplement: Document S1. Figures S1–S6, Tables S1–S3, and Supplemental Experimental Procedures [file mmc1.pdf]

Molecular Cell, *Volume 46*

Supplemental Information

Localized Dimerization and Nucleoid Binding Drive Gradient Formation by the Bacterial Cell Division Inhibitor MipZ

Daniela Kiekebusch, Katharine A. Michie, Lars-Oliver Essen, Jan Löwe, and Martin Thanbichler

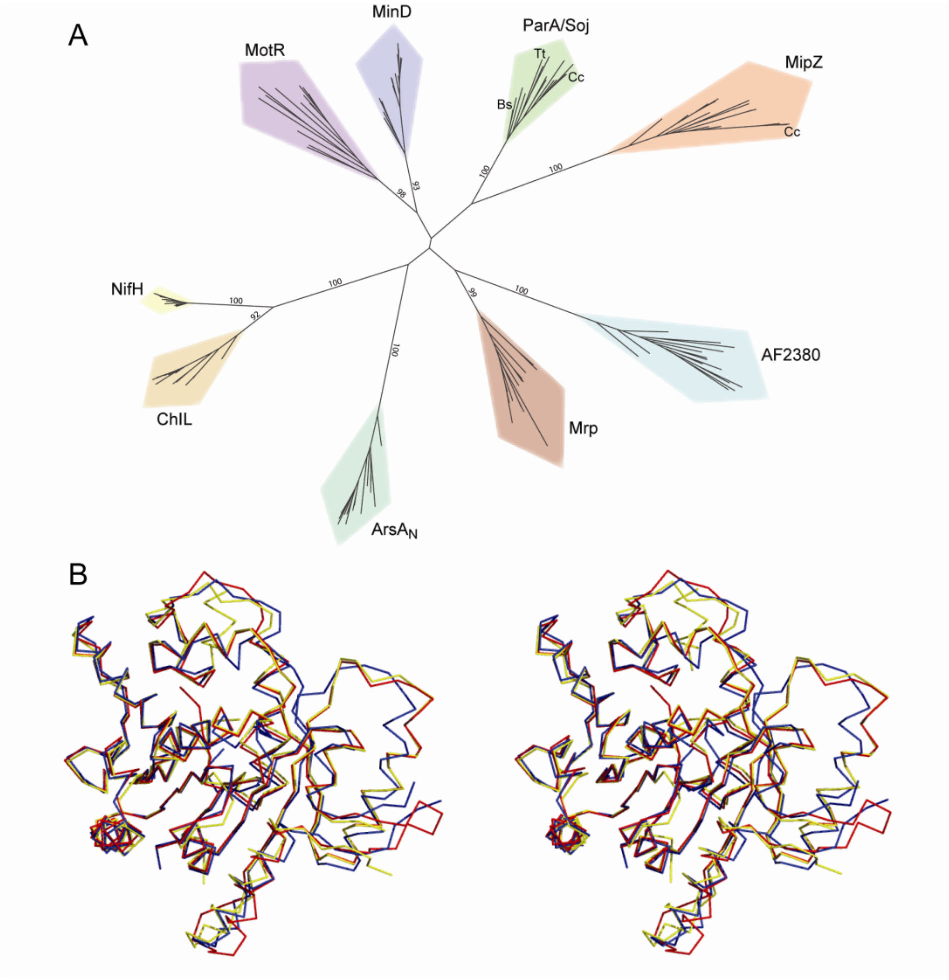

Figure S1. Phylogenetic and structural analysis of MipZ, Related to Figure 1.
(A) Evolutionary relationship between different Mrp/MinD ATPase family members. Protein sequences of 15 representative members from each subfamily were aligned with MAFFT v6 (Katoh et al., 2002). Phylogenetic relationships were determined using the maximum likelihood method as implemented in RaxML (Stamatakis et al., 2008) and visualized with the help of iTOL (Letunic and Bork, 2007). Numbers indicate bootstrap values. For ArsA orthologues, only the N-terminal domain was used for the alignment. Similar results were obtained with the C-terminal region (data not shown). Abbreviations: Cc (*Caulobacter crescentus*), Tt (*Thermus thermophilus*), Bs (*Bacillus subtilis*).
(B) Overlay of MipZ in different nucleotide states. Shown are the backbones of MipZ monomers in crystal form 1 (red) and crystal form 2 (yellow) as well as chain A of the MipZ dimer (blue).

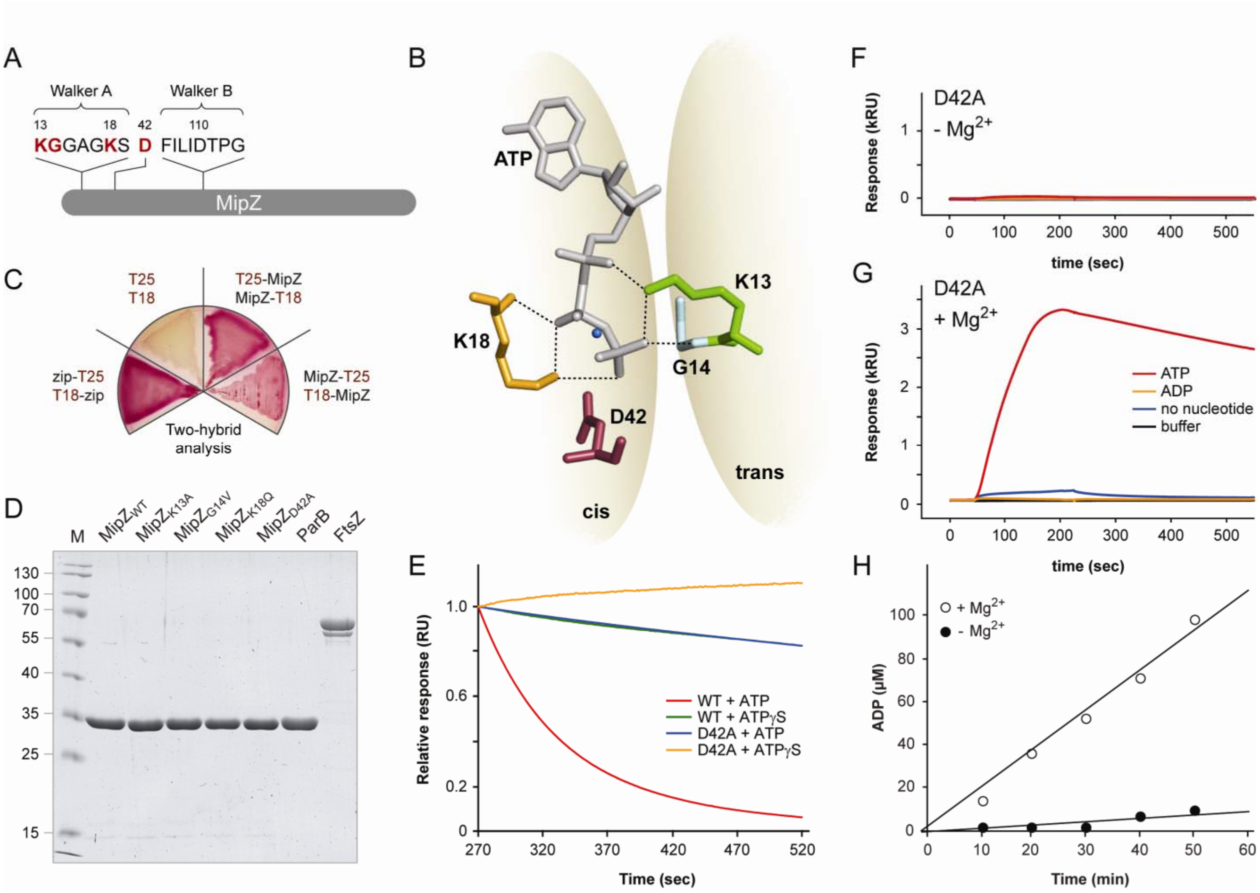

Figure S2. Mutational and biochemical analysis of MipZ dimerization, Related to Figure 2.

(A) Schematic of MipZ, showing the location of the Walker A/B motives and the catalytically important D42 residue. Numbers indicate the positions of residues in the MipZ polypeptide chain. The amino acids mutated in this study are highlighted in red.

(B) Structure of the MipZ catalytic site. Selected hydrogen bonds are indicated by dotted lines. The orientation of the D42 residue was approximated by mutating A42 in the crystal structure of the MipZ-D42A dimer (PDP 2XJ9) using PyMOL (DeLano Scientific LLC). Only one of the two catalytic sites in the MipZ dimer is shown. The two subunits are designated cis and trans.

(C) Detection of MipZ dimerization by bacterial two-hybrid analysis. Two-hybrid reporter strain *E. coli* BTH101 was transformed with plasmids encoding fusions of MipZ or the yeast GCN4 leucin-zipper region (zip) to the T25 and T18 fragments of *Bordetella pertussis* adenylate cyclase (see Supplemental Material). Transformants were restreaked on indicator plates. Interaction between the two adenylate cyclase fragments is evidenced by the formation of red colonies.

(D) Purified proteins. Solutions containing the indicated proteins (3 µg/lane) were applied to an 11% SDS-polyacrylamide gel. After electrophoresis, proteins were stained with Coomassie Blue.

(E) Surface plasmon resonance (SPR) analysis of dimer dissociation. MipZ or MipZ-D42A (3500 RU) were immobilized on a sensor chip and allowed to react with the respective soluble protein (6 µM) in the presence of 1 mM ATP or ATPγS. After the binding phase, the chip was washed with buffer lacking protein and nucleotides to follow the dissociation reactions. Data were normalized to facilitate visual comparison. Fitting of the transients to a single-exponential equation yielded the following dissocation rate constants (k_{off}): WT+ATP (0.83 min^{-1}), WT+ATPγS (0.18 min^{-1}), D42A+ATP (0.067 min^{-1}) and D42A+ATPγS (not measurable). Note that the curves for WT+ATP and D42A+ATP were taken from Fig. 2C.

(F and G) Effect of Mg^{2+} on dimer formation. MipZ-D42A (7000 RU) was immobilized on an SPR sensor chip and tested for homotypic interaction with soluble MipZ-D42A (0.5 µM) in buffer BC containing the indicated nucleotides (1 mM) and no (F) or 20 mM (G) EDTA. Mg^{2+}-dependent dimerization was also observed for wild-type MipZ (data not shown).

(H) Influence of Mg^{2+} on MipZ ATPase activity. MipZ (6 µM) was incubated with 1 mM [α-^{32}P]-ATP in buffer P containing no or 10 mM EDTA. Samples were taken at regular intervals and analyzed for the amount of ADP generated. Data represent the averages of two experiments.

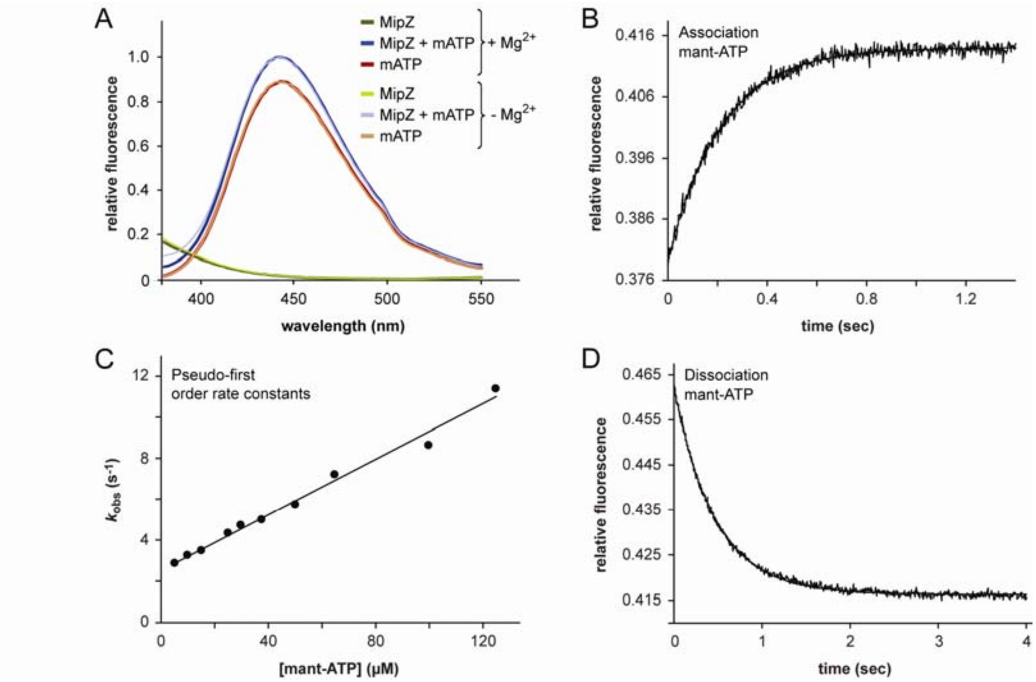

Figure S3. Steady-state and transient kinetic analysis of nucleotide binding to MipZ, Related to Table 2.

(A) Fluorescence emission spectra of mant-ATP. MipZ (1 µM) and/or mant-ATP (60 µM) were mixed in buffer BC containing no (+ Mg^{2+}) or 10 mM (- Mg^{2+}) EDTA. Emission spectra were recorded in a PC1 photon-counting spectrofluorimeter (ISS) using fluorescence resonance energy transfer (FRET) and an excitation wavelength of 290 nm.

(B) Association of MipZ with mant nucleotides. Nucleotide binding did not change the intrinsic tryptophan fluorescence of MipZ (data not shown), necessitating the use of ADP and ATP analogues that carried a fluorescent methylanthraniloyl (mant) group attached to the 2'- or 3'-hydroxy group of the ribose moiety. Due to the bulkiness of the mant group and a critical role of these hydroxy groups in MipZ dimerization, the modified nucleotides are predicted to block dimer formation, consistent with their failure to be hydrolyzed by MipZ or mediate MipZ self-association in the SPR assay (data not shown). This circumstance allowed us to focus the measurements on the initial nucleotide binding step without interference from subsequent reactions. Interaction of the mant group with the protein surface led to a marked increase in fluorescence (panel A). Consistent with the easy accessibility of the nucleotide-binding site and the lack of major conformational changes, the transients recorded after rapid mixing of MipZ with mant nucleotides could be fitted to single-exponential equations, indicating single-step binding reactions. Shown is a typical curve representing the change in mant fluorescence upon binding of mant-ATP (30 µM) to MipZ (1 µM). Fitting of the data yielded a pseudo-first order rate constant (k_{obs}) of 4.7 s^{-1}.

(C) Determination of the second-order association rate constants (k_{on}). The pseudo-first order rate constants for the association of MipZ with mant nucleotides showed a linear dependence on the nucleotide concentration used, with the slope of the fitted line determining the second-order rate constant of the association reaction. Shown is a representative graph in which the k_{obs} values measured for mant-ATP were plotted against the corresponding mant-ATP concentrations. Fitting of the data to the linear function k_{obs}=k_{on}[mant-ATP]+k_{off} yielded a value of 6.8 x 10^4 $M^{-1}s^{-1}$ for k_{on}.

(D) Dissociation of mant nucleotides from MipZ. In order to determine the dissociation rate constants (k_{off}), we monitored the displacement of mant-labeled nucleotides from MipZ by an excess of unlabeled nucleotide. All transients obtained could be fitted to single-exponential functions. Shown is a typical curve representing the change in mant fluorescence upon dissociation of mant-ATP (30 µM) from MipZ (1 µM) in the presence of 2 mM ATP. Fitting of the data yielded a dissociation rate constant (k_{off}) of 2.1 s^{-1}.

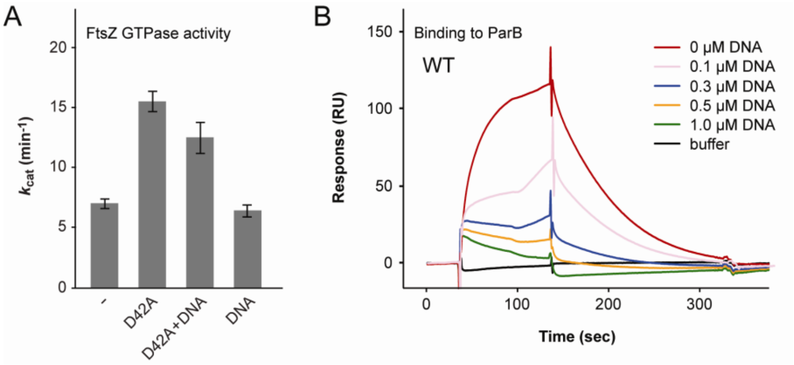

Figure S4. Effect of DNA on the MipZ-FtsZ and MipZ-ParB interaction, Related to Figure 3.

(A) Influence of DNA on the stimulation of FtsZ GTPase activity by MipZ. The GTPase activity of FtsZ (3 µM) was followed in the presence of 2 mM [α-^{32}P]-GTP and 3 mM ATP. The reactions were supplemented with 6 µM MipZ-D42A and/or 0.2 µM plasmid DNA (pMT428; 3.6 kb) as indicated. Data represent the mean of three independent measurements (± SD).

(B) Inhibitory effect of DNA on the interaction between MipZ and ParB. ParB (200 RU) was immobilized on an SPR sensor chip and allowed to react with wild-type MipZ protein (0.5 µM) that had been preincubated with 2 mM ATP and the indicated concentrations of a 26-bp double-stranded oligonucleotide (ran2). Note that injection of the DNA fragment alone did not produce any response (data not shown).

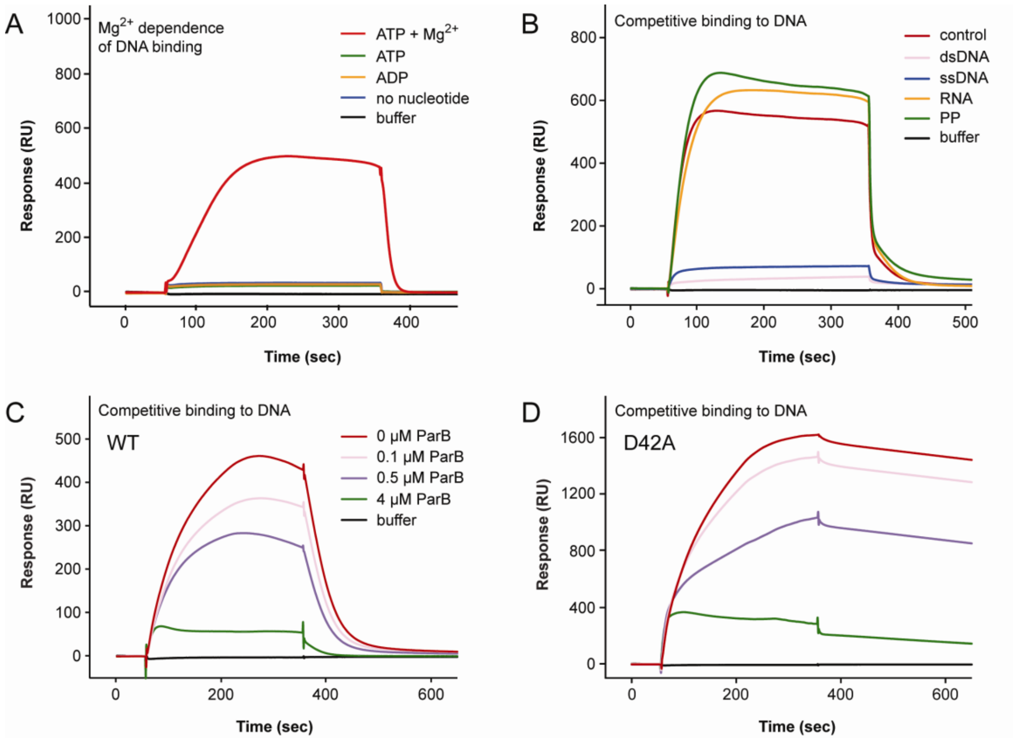

Figure S5. Surface plasmon resonance (SPR) analysis of the interaction between MipZ and DNA, Related to Figure 4.

(A) Effect of Mg^{2+} on the DNA-binding activity of MipZ. A double-stranded oligonucleotide (ran2, 700 RU) was immobilized on an SPR sensor chip and probed with MipZ (1 µM). The binding reactions were performed in buffer BC containing the indicated nucleotide (2 mM) and no (ATP + Mg^{2+}) or 10 mM (no nucleotide, ADP, ATP) EDTA, respectively.

(B) DNA-binding specificity of MipZ. A double-stranded oligonucleotide (ran2, 500 RU) was immobilized on an SPR sensor chip and allowed to react with MipZ protein (1 µM) that had been preincubated with 2 mM ATP and a double-stranded oligonucleotide (ran2, 6 µM) (dsDNA), a single-stranded oligonucleotide (top strand of ran2, 6 µM) (ssDNA), an oligoribonucleotide (with a sequence analogous to the top strand of ran2, 6 µM) (RNA) or polyphosphate (25 µg/ml) (PP), respectively. A reaction performed in the absence of any competitor served as a control. Note that consistent with the results of this analysis, only dsDNA and ssDNA can stimulate the ATPase activity of MipZ (compare Fig. 3G; data not shown).

(C) Inhibitory effect of ParB on the interaction between MipZ and DNA. A double-stranded oligonucleotide (ran2, 500 RU), immobilized on an SPR sensor chip, was probed with MipZ protein (0.5 µM) that had been preincubated with 2 mM ATP and the indicated concentrations of ParB. Note that ParB alone did not interact with the immobilized DNA fragment under the experimental conditions used (data not shown). The dissociation rate constant determined for the reaction without ParB was 1.5 min^{-1}.

(D) As for (C), but using MipZ-D42A.

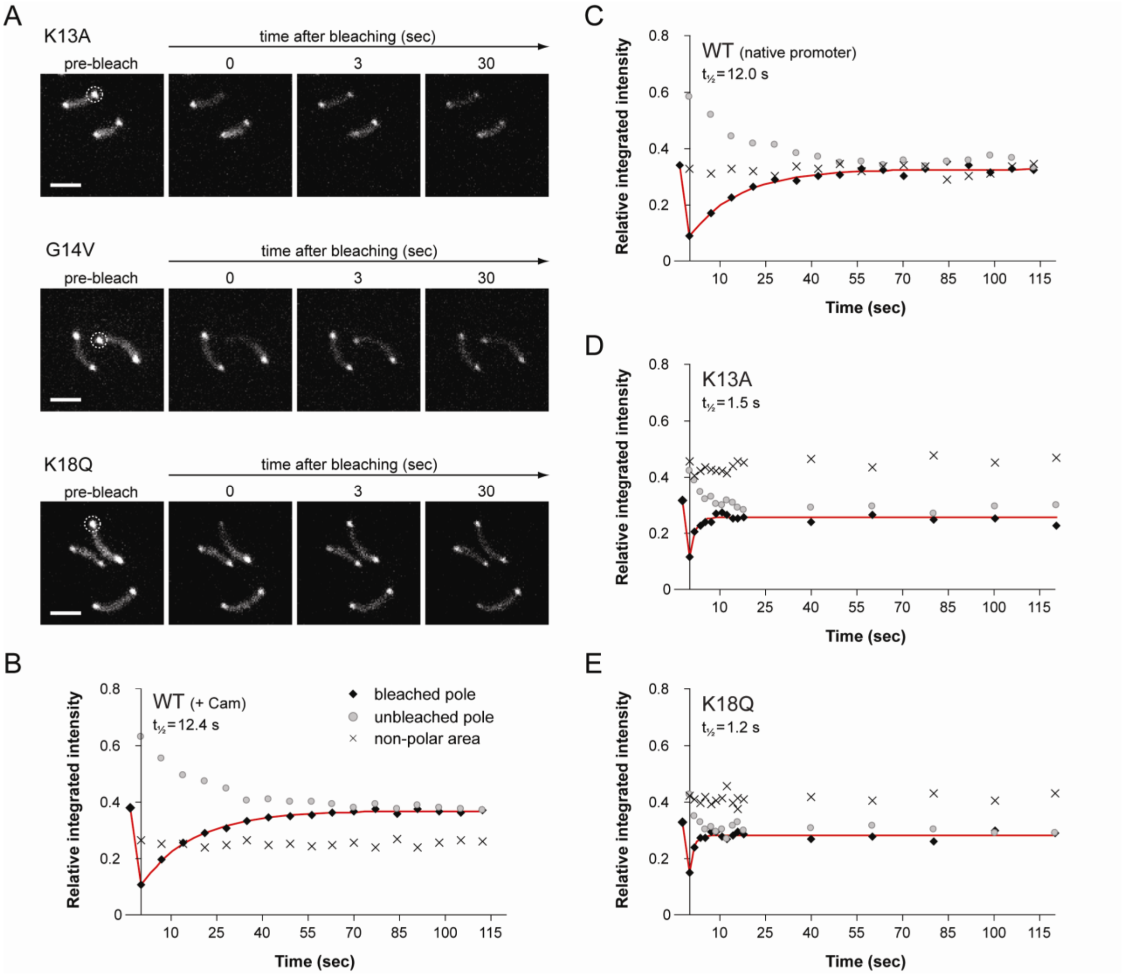

Figure S6. Fluorescence-recovery-after-photobleaching analysis of MipZ mobility, Related to Figure 5.

(A) Representative images of FRAP experiments on cells producing mutant MipZ-YFP fusions. Cells of strains DK2 (K13A), MT178 (G14V) and MT179 (K18Q), which express wild-type *mipZ* under the control of the ammonium-repressible P_{nrtX} promoter and the indicated *mipZ-eyfp* allele under the control of the xylose-inducible P_{xyl} promoter, were shifted from $M2G_N$ to M2G medium containing 0.3% xylose and cultivated for another 4 h to deplete wild-type MipZ and synthesize the fusion protein instead. Single polar regions were photo-bleached, and recovery was followed by fluorescence microscopy (bars: 2 µm). The bleached regions are highlighted by circles.

(**B**) Cells of strain MT177 (Δ*mipZ* P_{nrtX}::*mipZ* P_{xyl}::*mipZ-eyfp*) were grown as described in (A). Chloramphenicol (20 µg/ml) was added to the growing culture 25 min prior to harvest as well as to the agarose pads used for the FRAP experiments. Fitting of the data to a single exponential equation yielded a recovery half-time of 12.4 s (± 0.5 s).

(C) Cells of strain MT97 (*mipZ-eyfp*) were grown in M2G medium and subjected to FRAP analysis. The fitted curve corresponds to a recovery half-time of 12.0 s (± 1.2 s).

(D and E) Cells of strains (D) DK2 (Δ*mipZ* P_{nrtX}::*mipZ* P_{xyl}::*mipZ*$_{K13A}$-*eyfp*) and (E) MT179 (Δ*mipZ* P_{nrtX}::*mipZ* P_{xyl}::*mipZ*$_{K18Q}$-*eyfp*) were grown as described in (A) and subjected to FRAP analysis. Fitting of the data to a single-exponential equation yielded recovery half-times of 1.5 s (± 0.3 s) (K13A) and 1.2 s (± 0.2 s) (K18Q), respectively. Details on the experimental procedures and data analysis are described in the legend to Fig. 6.

Table S1. Plasmids, Related to the Experimental Procedures

Plasmid	Genotype/Description	Reference/Source
**Basic plasmids**		
pAMIOL-4	Plasmid for integrating genes at the *idhA* locus of CB15N, $Gent^R$	A. Möll (unpublished)
pBAD24-CB	Plasmid for the expression of genes in *E. coli* under the control of P_{BAD}, Amp^R	Möll & Thanbichler, 2009
pBluescript II KS(+)	Plasmid for general cloning purposes, Amp^R	Stratagene
pET21a(+)	Plasmid for overproduction of His-tagged proteins in *E. coli*, Kan^R	Novagen
pGADT7	Plasmid for creating N-terminal fusions to the transactivation domain of the yeast GAL4 protein	Clontech
pKT25	Plasmid used for creating N-terminal fusions to the T25 fragment of the catalytic domain of adenylate cyclise from *Bordetella pertussis*, Kan^R	Karimova et al., 1998
pHis17	Plasmid for overproduction of His-tagged proteins in *E. coli*, Amp^R	B. Miroux (LMB, unpublished)
pKNT25	Plasmid used for creating C-terminal fusions to the T25 fragment of the catalytic domain of adenylate cyclise from *Bordetella pertussis*, Kan^R	Karimova et al., 1998
pKT25-zip	pKT25 derivative harboring the leucine zipper of GCN4 fused to the T25 fragment of the catalytic domain of adenylate cyclase from *Bordetella pertussis*, Kan^R	Karimova et al., 1998
pMT329	Plasmid for expressing *cerulean-parB* under the control of P_{tet}, $Gent^R$	Thanbichler & Shapiro, 2006
pUT18	Plasmid used for creating C-terminal fusions to the T18 fragment of the catalytic domain of adenylate cyclise from *Bordetella pertussis*, Amp^R	Karimova et al., 1998
pUT18C	Plasmid used for creating N-terminal fusions to the T18 fragment of the catalytic domain of adenylate cyclise from *Bordetella pertussis*, Amp^R	Karimova et al., 1998
pUT18C-zip	pUT18C derivative harboring the leucine zipper of GCN4 fused to the T18 fragment of the catalytic domain of adenylate cyclase from *Bordetella pertussis*, Amp^R	Karimova et al.,1998
pVCERN-3	Plasmid for integrating N-terminal fusions to the cyan fluorescent protein Cerulean at the chromosomal *vanA* locus, Rif^R	Thanbichler et al., 2007
**Plasmids used in this study**		
pDK2	pBAD24-CB bearing *mipZ-eyfp*	This study
pDK3	pBAD24-CB bearing $mipZ_{D42A}$-*eyfp*	This study
pDK4	Plasmid for integrating $mipZ_{K13A}$-*eyfp* at the chromosomal *xylX* locus	This study
pDK10	pET21a bearing $mipZ_{K13A}$	This study
pDK14	pGADT7 bearing *mipZ*	This study
pDK22	pGADT7 bearing $mipZ_{D42A}$	This study
pDK23	pGADT7 bearing $mipZ_{G14V}$	This study
pDK24	pGADT7 bearing $mipZ_{K18Q}$	This study
pDK25	pGADT7 bearing $mipZ_{K13A}$	This study
pDK34	pKT25 bearing *mipZ*	This study
pDK35	pKNT25 bearing *mipZ*	This study
pDK36	pUT18 bearing *mipZ*	This study
pDK37	pUT18C bearing *mipZ*	This study
pHis17mipZ	pHis17 bearing wild-type *mipZ*	This study
pHis17mipZD42A	pHis17 bearing $mipZ_{D42A}$	This study
pMT151	Plasmid for integrating *mipZ-eyfp* at the chromosomal *xylX* locus	Thanbichler & Shapiro, 2006
pMT174	Plasmid for integrating $mipZ_{G14V}$-*eyfp* at the chromosomal *xylX* locus	This study
pMT183	pET21a (+) bearing *mipZ*	Thanbichler & Shapiro, 2006
pMT209	Plasmid for integrating $mipZ_{K18Q}$-*eyfp* at the chromosomal *xylX* locus	This study
pMT219	pET21a (+) bearing *ftsZ*	Thanbichler & Shapiro, 2006
pMT314	pET21a (+) bearing *parB*	Thanbichler & Shapiro, 2006
pMT362	Plasmid for integrating $mipZ_{D42A}$-*eyfp* at the chromosomal *xylX* locus	Thanbichler & Shapiro, 2006
pMT413	pET21a (+) bearing $mipZ_{G14V}$	This study
pMT414	pET21a (+) bearing $mipZ_{K18Q}$	This study
pMT415	pET21a (+) bearing $mipZ_{D42A}$	Thanbichler & Shapiro, 2006
pMT428	pBluescript II KS(+) bearing the chromosomal P_{gidA} region	This study
pMT977	pAMIOL-4 bearing *cfp-parB*	This study

Table S2. Strains, Related to the Experimental Procedures

Strain	Genotype/Description	Reference/Source
E. coli		
TOP10	General cloning strain	Invitrogen
DH101	Bacterial two-hybrid reporter strain	Euromedex
Rosetta2(DE3)pLysS	Strain for protein overproduction	Novagen
S. cerevisiae		
AH109	Yeast two-hybrid reporter strain	Clontech
C. crescentus		
MT97	CB15N *mipZ-eyfp*	Thanbichler & Shapiro, 2006
MT148	CB15N *mipZ-eyfp parB*::P_{xylX}-*parB*	Thanbichler & Shapiro, 2006
MT157	CB15N Δ*mipZ* P_{nrtX}::P_{nrtX}-*mipZ xylX*::*mipZ*	Thanbichler & Shapiro, 2006
MT177	CB15N Δ*mipZ* P_{nrtX}::P_{nrtX}-*mipZ* P_{xylX}::P_{xylX}-*mipZ-eyfp*	Thanbichler & Shapiro, 2006
MT178	CB15N Δ*mipZ* P_{nrtX}::P_{nrtX}-*mipZ* P_{xylX}::P_{xylX}-*mipZ*$_{G14V}$-*eyfp*	This study
MT179	CB15N Δ*mipZ* P_{nrtX}::P_{nrtX}-*mipZ* P_{xylX}::P_{xylX}-*mipZ*$_{K18Q}$-*eyfp*	This study
MT197	CB15N Δ*mipZ* P_{nrtX}::P_{nrtX}-*mipZ* P_{xylX}::P_{xylX}-*mipZ*$_{D42A}$-*eyfp*	Thanbichler & Shapiro, 2006
MT303	CB15N P_{iol}::P_{iol}-*cfp-parB*	This study
DK2	CB15N Δ*mipZ* P_{nrtX}::P_{nrtX}-*mipZ* P_{xylX}::P_{xylX}-*mipZ*$_{K13A}$-*eyfp*	This study
DK3	CB15N Δ*mipZ* P_{nrtX}::P_{nrtX}-*mipZ* P_{xylX}::P_{xylX}-*mipZ*$_{K13A}$-*eyfp* P_{iol}::P_{iol}-*cfp-parB*	This study
DK5	CB15N Δ*mipZ* P_{nrtX}::P_{nrtX}-*mipZ* P_{xylX}::P_{xylX}-*mipZ*$_{G14V}$-*eyfp* P_{iol}::P_{iol}-*cfp-parB*	This study
DK6	CB15N Δ*mipZ* P_{nrtX}::P_{nrtX}-*mipZ* P_{xylX}::P_{xylX}-*mipZ*$_{K18Q}$-*eyfp* P_{iol}::P_{iol}-*cfp-parB*	This study
DK7	CB15N Δ*mipZ* P_{nrtX}::P_{nrtX}-*mipZ* P_{xylX}::P_{xylX}-*mipZ*$_{D42A}$-*eyfp* P_{iol}::P_{iol}-*cfp-parB*	This study

Table S3. Oligonucleotides, Related to the Experimental Procedures.

Restriction sites are indicated by capital letters.

Oligonucleotide	Sequence
CC2165-G14V	TTTTCATatggccgaaacgcgcgttatcgtcgtcggcaacgagaaggtcggcgctggc
CC2165-K13A-QCM1	cgtcgtcggcaacgaggcgggcggcgctggcaagtcgacc
CC2165-K13A-QCM2	ggtcgacttgccagcgccgcccgcctcgttgccgacgacg
CC2165-K18Q-1	cagtcgaccatcgccgtgcacctcgtc
CC2165-K18Q-2	gccagcgccgcccttctcgttgccg
CC2165-rev	ATGAATTCtcattactgcgccgccagcatcgtctcgcc
CC2165-rev2	TTGAGCTCctgcgccgccagcatcgtctcgcc
CC2165-revHindIII	CCGCAAGCTTgtcgacggcctgcgccgccagcatcgtctcgcc
CC2165-uni2	TTTTCATatggccgaaacgcgcgttatcgtcg
mipZ-BACTH-for	TTTTGGATCCCatggccgaaacgcgcgttatcgtcg
mipZ-KNT25-rev	AAAAGAATTCGActgcgccgccagcatcgtctcgcc
mipZ-KT25-rev	AAAAGAATTCTTTActgcgccgccagcatcgtctcgcc
mipZ-UT18C-rev	AAAAGAATTCGATTActgcgccgccagcatcgtctcgcc
PgidA-1	TAGAATTCcggatgtgatcgaggccgagggtgttc
PgidA-2	TAGGATCCtcaggcgagcgggtcgatgtgactcat
Pxyl-1	cccacatgttagcgctaccaagtgc

SUPPLEMENTAL EXPERIMENTAL PROCEDURES

Media and growth conditions

All *C. crescentus* strains used in this study were derived from the synchronizable wild-type strain CB15N (NA1000) (Evinger and Agabian 1977). They were grown at 28°C in M2G (Ely, 1991) or $M2G_N$ (Thanbichler and Shapiro, 2006) minimal medium, supplemented with antibiotics at the following concentrations (µg/ml; liquid/solid medium) when appropriate: spectinomycin (25/50), streptomycin (-/5), gentamicin (0.5/5), kanamycin (5/25).

E. coli strains TOP10 and Rosetta2(DE3)/pLysS (Invitrogen) were cultivated at 37°C in LB medium (Carl Roth, Germany) supplemented with antibiotics at the following concentrations (µg/ml; liquid/solid medium): ampicillin (50/100), chloramphenicol (20/30), kanamycin (30/50), cephalexin (5/-). When appropriate, arabinose was added to a final concentration of 0.1 %.

E. coli BTH101 (Karimova et al. 1998) transformed with complementary two-hybrid plasmids was routinely grown at 37°C in LB broth containing 200 µg/ml ampicillin and 50 µg/ml kanamycin. For interaction analyses, cells were streaked onto MacConkey agar plates (Carl Roth, Germany) supplemented with 1 % maltose and incubated at 30°C for 24 h.

Yeast strain AH109 was routinely grown in YPAD medium at 30 °C. Transformants bearing two-hybrid plasmids were cultivated in SD/-Trp/-Leu minimal medium following the Yeast Protocols Handbook provided by the manufacturer (Clontech).

To study the effects of different mutations on the activity of MipZ, strains MT177 (WT), DK2 (K13A), MT178 (G14V), MT179 (K18Q) and MT197 (D42A), which express wild-type *mipZ* under the control of the NH_4^+-repressible P_{nrtX} promoter and the indicated *mipZ-eyfp* allele under the control of the xylose-inducible P_{xyl} promoter, were shifted from $M2G_N$ medium (- NH_4^+) to M2G medium (+ NH_4^+) supplemented with 0.3% xylose and incubated for 4-8 h. Strains carrying *cerulean-parB* under the control of the inositol-inducible P_{iol} promoter (DK3, DK5, DK6 and DK7) were induced for 2 h with 0.3% myo-inositol before microscopy.

To analyze the unspecific DNA-binding activity of MipZ, *E. coli* TOP10 was transformed with plasmid pDK2 (P_{BAD}-*mipZ-eyfp*) or pDK3 (P_{BAD}-*mipZ*$_{D42A}$-*eyfp*) and grown for 2 h in the presence of cephalexin and arabinose to block cell division and activate synthesis of the fusion proteins, respectively. Thirty minutes prior to analysis, chloramphenicol (100 µg/ml) was added to induce nucleoid condensation. DNA was stained with DAPI, and the cells were visualized by microscopy.

Plasmid construction

Plasmids encoding fluorescent protein fusions. To generate **pMT174**, *mipZ* was PCR-amplified with the mutagenic primers CC2165-G14V and CC2165-rev2. The reaction product containing the mutant *mipZ*$_{G14V}$ allele was cut with NdeI and SacI and ligated into plasmid pMT151, an integrative plasmid carrying a *mipZ-eyfp* fusion, whose wild-type *mipZ* gene had been released by restriction with the same enzymes. Plasmid **pMT209** was constructed as follows: Inverse PCR was performed on plasmid pMT151 using primers CC2165-K18Q-1 and CC2165-K18Q-2 to generate the mutant *mipZ*$_{K18Q}$ allele. After phosphorylation and self-ligation of the PCR product, the resulting plasmid was cut with NdeI and SacI. The fragment containing *mipZ*$_{K18Q}$ was then ligated into plasmid pMT151 whose wild-type *mipZ* gene had been released by restriction with NdeI and SacI. To generate plasmid **pMT977**, the *cerulean* gene was released from pVCERN-3 by restriction with NdeI and KpnI. In parallel, the *parB* gene was isolated from pMT329 by restriction with KpnI and XbaI. The two fragments were combined and ligated into pAMIOL-4 cut with AseI and NheI. Plasmid **pDK4** was constructed by site-directed mutagenesis of pMT151 using primers CC2165-K13A-QCM1 and CC2165-K13A-QCM2. The *mipZ*$_{K13A}$ gene was released by restriction of the PCR product with NdeI and SacI and ligated into pMT151 cut with the same enzymes. To generate plasmids **pDK2** and **pDK3**, the *mipZ-yfp* and *mipZ*$_{D42A}$-*yfp* fusions were released from plasmids pMT151 and pMT362, respectively, by restriction with XhoI, subsequent blunting with T4 DNA polymerase, and restriction with NdeI. The fragments were then ligated into pBAD24-CB that had been cut with HindIII, blunted with T4 DNA polymerase, and then cut with NdeI.

Plasmids for protein overproduction in *E. coli*. To construct plasmid pDK10, a fragment bearing *mipZ*$_{K13A}$ was PCR-amplified from pDK4 using primers Pxyl-1 and CC2165-revHindIII. The product was cut with NdeI and HindIII and ligated into equally treated pET21a(+). For the construction of **pMT413** and **pMT414**, the *mipZ*$_{G14V}$ and *mipZ*$_{K18Q}$ alleles were PCR-amplified from suitable templates using primers CC2165-uni2 and CC2165-rev. The PCR products were cut with NdeI and NcoI and the 5' fragments released were ligated into equally treated pMT183, respectively. To generate **pHis17mipZ**, the *mipZ* gene from *C. crescentus* (ATCC 19089D) was cloned into pHis17, generating a hybrid that encodes a C-terminally hexahistidine-tagged protein. For the construction of plasmid **pHis17mipZD42A**, overlap extension PCR was used to generate the mutant *mipZ*$_{D42A}$ allele, and the resulting PCR product was cloned into pHis17.

Plasmids for bacterial two-hybrid analysis. To construct **pDK34**, *mipZ* was PCR-amplified from pMT183 using primers mipZ-BACTH-for and mipZ-KT25-rev. The reaction product was cut with BamHI and EcoRI and ligated into equally treated pKT25. Plasmids **pDK35** and **pDK36** were generated by PCR

amplification of *mipZ* from pMT183 with primers mipZ-BACTH-for and mipZ-KNT25-rev, restriction of the reaction product with BamHI and EcoRI, and subsequent ligation of the fragments into equally cut pKNT25 and pUT18, respectively. To construct **pDK37**, *mipZ* was PCR-amplified from pMT183 using primers mipZ-BACTH-for and mipZ-UT18C-rev. The products were digested with BamHI and EcoRI and ligated into pUT18C treated with the same enzymes.

Plasmids for yeast one-hybrid analysis. To generate plasmid **pDK14,** *mipZ* was PCR-amplified from genomic DNA with primers CC2165-uni2 and CC2165-rev. The product was cut with NdeI and EcoRI and ligated into equally digested pGADT7. Plasmids **pDK22**, **pDK23**, **pDK24** and **pDK25** were constructed in the same way using plasmids bearing mutant alleles of *mipZ* as PCR templates.

Other plasmids. To construct plasmid **pMT428**, P_{gidA} was PCR-amplified from genomic DNA using primers PgidA-1 and PgidA-2. The product was cut with EcoRI and BamHI and ligated into equally digested pBluescript II-KS(+).

Strain construction

C. crescentus was transformed by electroporation (Ely, 1991). Proper chromosomal integration of non-replicating vectors was verified by colony PCR (Thanbichler et al., 2007). Generalized transduction using phage ΦCr30 was performed as described previously (Ely and Johnson, 1977).

Strains **MT112**, **MT113** and **DK1** were generated by integration of plasmids pMT174, pMT209 and pDK4, respectively, at the *xylX* locus of wild-type strain CB15N by single homologous recombination. To construct **MT178**, **MT179** and **DK2**, strain MT157 was transduced with ΦCr30 lysates of MT112, MT113 and DK1, thereby replacing the *mipZ* gene integrated at the *xylX* locus of MT157 with a $mipZ_{G14V}$-*eyfp*, $mipZ_{K18Q}$-*eyfp* or a $mipZ_{K13A}$-*eyfp* fusion, respectively. Strain **MT303** was created by integrating plasmid pMT977 at the *idhA* (CC1296) locus (Boutte et al. 2008) of wild-type strain CB15N. To generate strains **DK3**, **DK5**, **DK6** and **DK7**, the *cerulean-parB* fusion carried by strain MT303 was transferred to strains DK2, MT178, MT179 and MT197, respectively, by ΦCr30-mediated generalized transduction.

Protein expression and purification

For biochemical studies, hexahistidine-tagged forms of MipZ or its mutant derivatives as well as native ParB and FtsZ were overproduced in *E. coli* Rosetta2 (DE3)pLysS (Invitrogen) and purified as described previously (Thanbichler and Shapiro 2006).

The protein used for crystallizing apo-MipZ in crystal form 1 and the MipZ dimer was prepared as follows: *E. coli* strain BL21 AI (Invitrogen) was transformed with pHis17mipZ or pHis17mipZD42A and grown at 37˚C in 2xTY medium or, in the case for selenomethionine (SeMet)-labelled protein, in M9 minimal medium (Miller 1972) that was supplemented with amino acids and selenomethionine ten minutes prior to induction as described previously (van den Ent et al. 1999). At an OD_{600} of 0.8, protein synthesis was induced with the addition of 0.2% arabinose. After another 4 h of incubation, the cells were harvested by centrifugation, frozen, and stored at -70˚C.

To purify wild-type MipZ, the cells were lysed in 50 mM Tris/HCl (pH 8.0). The lysate was cleared by ultracentrifugation at 40,000 rpm in Beckman Ti45 rotor (4°C) and loaded on a HisTrap column (GE Healthcare). After a wash with 50 mM Tris/HCl/300 mM NaCl (pH 7.0), the protein was eluted with 50 mM Tris/HCl/ 300 mM imidazole (pH 7.0). Further purification involved ion-exchange chromatography on a HiTrap Q column (GE Healthcare), on which MipZ was not retained. The flow-through was concentrated in Vivaspin protein concentrators (10,000 MWCO) at 4˚C. Subsequently, the protein was applied to a Sephacryl S-200 gel filtration column (GE Healthcare) equilibrated in TEN buffer (20 mM Tris/HCl, pH 7.5, 1 mM EDTA, 1 mM sodium azide) containing 100 mM NaCl. The final sample was concentrated to 2.6 mg/mL and stored at -70˚C. In the case of the SeMet-labeled protein, a reducing agent (either DTT or TCEP) was added to all buffers throughout the purification.

$MipZ_{D42A}$ was purified in a similar manner, leaving out the ion-exchange chromatography step and using TEN buffer with 300 mM salt for gel filtration. Mass spectral analysis revealed a mass of 31598 Da for native MipZ, and 31918 Da for selenomethionine-labeled MipZ, indicating that both proteins lacked the first methionine. All seven remaining methionine residues were successfully labelled with selenium.

For the crystallization of apo-MipZ in crystal form 2, hexahistidine-tagged MipZ was purified from Rosetta2 (DE3)/pLysS transformed with pMT183 as described previously (Thanbichler and Shapiro, 2006).

Crystallization and structural analysis

For apo-MipZ in crystal form 1, crystallization conditions were screened using MipZ at 2.6 mg/mL plated into 200 nl sitting-well drops in 1440 standard conditions using a robotics facility (Stock et al. 2005). Only one condition (100 mM MES, 30% PEG 400, pH 6.5) was identified. Crystals of SeMet-substituted MipZ were obtained in the same condition with streak-seeding from the native MipZ crystals. Data sets for both proteins were obtained from crystals flash frozen in mother liquor. Data were collected at ID23-1, ESRF (Grenoble, France).

$MipZ_{D42A}$ was screened under the same 1440 standard conditions in the presence of 1 mM ATP or 1 mM ATPγS. Crystallization was observed in three buffers, which all contained ATPγS and 100 mM Tris (pH 8.5) with 18% - 20% ethanol. Data sets for $MipZ_{D42A}$ were

obtained from crystals flash frozen in a solution of 95% ethanol/50 mM Tris (pH 8.5), and collected at ID23-2, ESRF (Grenoble, France). For apo-MipZ in crystal form 2, 768 standard conditions were screened in 200 nl sitting drops using a Microsys 4004 Cartesian robotics system and MipZ at a concentration of 10 mg/ml. Crystals obtained in a buffer containing 100 mM Hepes (pH 7.5), 10% PEG 10000, 4% ethylene glycol and 30% glycerol were flash-frozen, and data were collected at ID23-1, ESRF.

All crystals were indexed and integrated using the MOSFLM package and further processed using the CCP4 package (Collaborative Computational Project, 1994). Refinement was performed using PHENIX (Adams et al., 2002; Adams et al., 2004) for apo-MipZ in crystal form 1 and $MipZ_{D42A}$ and REFMAC5 (Murshudov et al. 1997) for apo-MipZ in crystal form 2. The structure of apo-MipZ in crystal form 1 was solved by multiple-wavelength anomalous diffraction (MAD), while the structures of apo-MipZ in crystal form 2 and $MipZ_{D42A}$ were solved by molecular replacement.

For downstream analysis, sequences were aligned with ClustalW (Larkin et al., 2007) and secondary structural elements were identified using DSSP (Kabsch and Sander, 1983). 3D structural alignments and stereo superpositions were generated with Superpose V1.0 (Maiti et al., 2004), and surface electrostatic potentials were calculated using CCP4mg (Potterton et al., 2004).

Yeast one-hybrid analysis

Yeast reporter strain AH109 was transformed with plasmids encoding fusions of MipZ or its mutant variants to the GAL4 transactivation domain. Liquid β-galactosidase activity assays were performed following the protocol provided with the Matchmaker™ GAL4 Two Hybrid System 3 (Clontech).

Nucleotide hydrolysis assays

For ATPase activity assays, MipZ or its derivatives (6 µM) were preincubated for 10 min at 30°C in buffer P (50 mM Hepes/NaOH, pH 7.2, 50 mM KCl, 10 mM $MgCl_2$, 1 mM β-mercaptoethanol) supplemented with the indicated components. The reaction was started by addition of [α-^{32}P]-ATP (25 Ci/mmol) to a final concentration of 1 mM. At 10-min intervals, 2 µl samples were taken and immediately spotted onto PEI-cellulose F thin-layer chromatography plates (Merck). The plates were developed in a solvent system containing 0.5 M LiCl and 1 M formic acid, air-dried, and exposed to a storage phosphor screen (GE Healthcare). After scanning of the screen with a Storm 840 PhosphorImager (GE Healthcare), the amount of [α-^{32}P]-ADP present in the samples was quantified using ImageQuant 5.2 (Molecular Dynamics).

For GTPase assays, FtsZ (3 µM) was preincubated with MipZ or BSA (6 µM) and 1 mM ATP for 10 min at 30°C in buffer P. After starting the reaction by addition of [α-^{32}P]-GTP (25 Ci/mmol) to a final concentration of 2 mM, samples were taken and analyzed for the amount of [α-^{32}P]-GDP produced in the reaction as described above.

To analyze the concentration dependence of MipZ's ATPase activity, varying amounts of MipZ were incubated in buffer P containing 2 mM [α-^{32}P]-ATP and 0.3 mg/ml bovine serum albumin and assayed for hydrolytic activity as described above.

To determine the effect of ParB on MipZ's ATPase activity, varying amounts of MipZ were incubated with ParB (4.5 µM) and/or plasmid pMT428 (3.6 kb, 0.2 µM), which bears a chromosomal fragment containing all predicted *C. crescentus parS* sites in buffer P containing 2 mM [α-^{32}P]-ATP and assayed for hydrolytic activity as described above.

Transient kinetic analysis

Transient kinetic experiments were conducted at 30°C in a Hi-Tech Scientific SF-61MX stopped-flow spectrofluorimeter operated in FRET mode, using an excitation wavelength of 297 nm and a 360-nm emission cut-off filter. All solutions were prepared in buffer P (50 mM Hepes/NaOH, pH 7.2, 50 mM KCl, 10 mM $MgCl_2$, 1 mM β-mercaptoethanol). In general, 10-20 traces were averaged for each condition. The observed rate constants (k_{obs}) were determined by fitting of the data to a single-exponential equation using Kinetic Studio 2.00 (TgK Scientific). Secondary analysis was performed using Excel (Microsoft) or Origin 6.1 (OriginLab).

Surface plasmon resonance analysis

Biosensor experiments were performed on a Biacore T100 system at 25°C and a flow rate of 30 µl/min using buffer P2 (10 mM Hepes/NaOH, pH 7.2, 150 mM NaCl, 10 mM $MgCl_2$, 0.05 % Tween 20) or buffer BC (50 mM Hepes/NaOH, pH 7.2, 50 mM KCl, 5 mM $MgCl_2$). Proteins were dialyzed against the respective assay buffer before use. To analyze the binding of MipZ to dsDNA (in buffer P2), biotinylated double-stranded oligonucleotides (top strands: $parS_{WT}$, GAG-GCTTGTTTCACGTGAAACATCGG; $parS_{mt}$, GAGG-CTTGTTTGAGCTCAAACATCGG; ran1, GAGGCA-GACTAGATCTTCTAGTTCGG; ran2, GTCGACAG-AGTTCGCTAACATCGAGC) were immobilized on a streptavidin-coated Sensor Chip SA (GE Healthcare) according to the manufacturer`s recommendations. Self-association of MipZ and interaction of MipZ with ParB (in buffer BC) was examined using a Sensor Chip CM5 (GE Healthcare) to which MipZ or ParB was covalently attached via amine coupling as described by the manufacturer.

For self-association of MipZ, the reactions contained 0.5 µM protein and 1 mM ATP or ADP when appropriate. For the analysis of the MipZ-ParB or MipZ-DNA interactions, proteins were used at a concentration of 6 µM in the presence of 1 mM ADP or ATP, if not indicated otherwise. After the binding phase, the chip was washed with buffer lacking protein

and nucleotides to follow the dissociation reactions. The chip surface was regenerated by washing for 20 s with 7 mM NaOH (for protein-DNA interactions) or buffer BC containing 2 M NaCl (for protein-protein interactions). In all experiments, a mock-treated reference cell lacking immobilized ligand was used to correct for non-specific binding to the sensor chip surface. Primary data analysis was performed with the BIAevaluation software (GE Healthcare). Dissociation rate constants were determined by fitting of the response curves to a single-exponential equation using the Origin 6.1 software (OriginLab).

Size exclusion chromatography

Wild-type MipZ or MipZ-D42A (both at 2 mg/ml) were incubated for 2 min in a buffer (20 mM CHES, pH 10, 100 mM NaCl, 1 mM $MgCl_2$, 1 mM azide) containing either 1 mM ADP, ATP or ATPγS. The samples (25 µl) were spun in a microfuge for 1 min. Subsequently, the soluble fraction was injected onto a Superdex 200 Precision Column (2.4 ml; GE Healthcare) that was equilibrated in CHES buffer (20 mM CHES, 100 mM NaCl, 1 mM MgCl and 1 mM azide, pH 10) containing 0.1 mM ADP (for ADP-bound protein) or ATP (for ATP- and ATPγS-bound protein). Protein was eluted at a flow rate of 0.05 ml/min.

Electrophoretic mobility shift assays

The ability of MipZ to bind double-stranded DNA was assayed in the presence of 1.25 mM nucleotide (ADP, ATP or ATPγS) and 2.5 mM Mg^{2+}. Binding reactions were performed in 15 µl volumes containing 25 mM Tris/HCl (pH 8.0), 100 mM KCl, 10% glycerol and 50 µg/mL BSA. Each reaction contained 60 ng (23 nM) of a 282-bp PCR product, amplified from *Thermotoga maritima* chromosomal DNA using the primers CCGGAATTCCATATGCAGCTCAAGGCCGCTAT-TGAG and CGGGATCCTCAAGACTCGCCACCA-TCGC and protein at concentrations ranging from 270 nM to 5.6 µM. Reactions were incubated at room temperature for 20 min and then loaded on a 4-20% gradient polyacrylamide gel (Criterion TBE, Bio-Rad), which was run for 90 min at 4˚C and a constant voltage of 100 V in TBE buffer (89 mM Tris, 89 mM boric acid, 2 mM EDTA). DNA was stained with ethidium bromide before visualisation with UV light.

FRAP analysis

For FRAP analyses were performed on the same microscope setup equipped with a 488 nm-solid state laser and a 2D-VisiFRAP Galvo System multi-point FRAP module (Visitron Systems, Germany), using single 30-ms pulses at a laser power of 1%. Cells were transferred onto pads made of 1.5% agarose in M2G medium. After acquisition of a pre-bleach image and application of the laser pulse, fluorescence recovery was monitored at regular intervals over a period of at least two minutes. For each timepoint, the integrated fluorescence intensities of the whole cell, the bleached region and an equally sized unbleached region were measured using Metamorph 7.1.2. After background correction, the fluorescence intensities of the bleached and unbleached regions were divided by the whole cell intensity to correct for general photobleaching during the imaging process, and the average values of 17-29 cells were plotted. Recovery rates were determined with the SOLVER function of Microsoft Excel by fitting the data obtained for the bleached region to the single exponential function F(t) = A(1-e-k*t)+F0, where F(t) is the fluorescence at time t, A the maximum intensity, k the rate constant and F0 the relative fluorescence intensity at t=0 min. In all cases, fits with R2 ≥ 0.95 were obtained. Recovery half-times were calculated according to the equation t1/2 = ln(0.5)/-k.

Rotary shadowing electron microscopy

Phage ΦX174 RFII DNA (NEB) was digested with *Xho*I (NEB), extracted with phenol/ chloroform, and used at a final concentration of 100 pg μl^{-1}. $MipZ_{D42A}$ (at concentrations ranging from 150 nM to 5 µM) was incubated with ATPγS and/or DNA for 10 min at room temperature. The samples were then dialyzed for 1 h at 4°C against 150 mM ammonium bicarbonate, 40% glycerol, 100 µM ATP, using 10000 MWCO micro dialysis units (Pierce). 5-µl samples were spread onto freshly cleaved mica sheets and rotary-shadowed at an oblique angle of 8˚ and maximum rotation speed for 20 sec in a freeze fracture and etching system (Cressington Scientific, PA), using a platinum electron-beam metal evaporation gun operated at 80 mA/2.2 kV. Immediately following platinum coating, two sequential 16-sec depositions of carbon (100 mA/2.5 kV) were made at an angle of 90˚ to the rotating sample for increased structural support. After removal from the vacuum chamber, samples were floated off on water and transferred onto copper grids. Images were taken with a Philips EM 208 transmission electron microscope at 80 kV and magnifications between 79,000 and 180,000 x.

SUPPLEMENTAL REFERENCES

Adams, P.D., Gopal, K., Grosse-Kunstleve, R.W., Hung, L.W., Ioerger, T.R., McCoy, A.J., Moriarty, N.W., Pai, R.K., Read, R.J., Romo, T.D., Sacchettini, J.C., Sauter, N.K., Storoni, L.C., and Terwilliger, T.C. (2004) Recent developments in the PHENIX software for automated crystallographic structure determination. J Synchrotron Radiat *11*, 53-55.

Adams, P.D., Grosse-Kunstleve, R.W., Hung, L.W., Ioerger, T.R., McCoy, A.J., Moriarty, N.W., Read, R.J., Sacchettini, J.C., Sauter, N.K., and Terwilliger, T.C. (2002) PHENIX: building new software for automated crystallographic structure determination. Acta Crystallogr D Biol Crystallogr *58*, 1948-1954.

Boutte, C.C., Srinivasan, B.S., Flannick, J.A., Novak, A.F., Martens, A.T., Batzoglou, S., Viollier, P.H., and Crosson, S. (2008) Genetic and computational identification of a conserved bacterial metabolic module. PLoS Genet *4*, e1000310.

Collaborative Computational Project, Number 4 (1994) The CCP4 suite: programs for protein crystallography. Acta Crystallogr D Biol Crystallogr *50*, 760-763.

Ely, B. (1991) Genetics of *Caulobacter crescentus*. Methods Enzymol *204*, 372-384.

Evinger, M. and Agabian, N. (1977) Envelope-associated nucleoid from *Caulobacter crescentus* stalked and swarmer cells. J Bacteriol *132*, 294-301.

Kabsch, W., and Sander, C. (1983). Dictionary of protein secondary structure: pattern recognition of hydrogen-bonded and geometrical features. Biopolymers *22*, 2577-2637.

Karimova, G., Pidoux, J., Ullmann, A., and Ladant, D. (1998) A bacterial two-hybrid system based on a reconstituted signal transduction pathway. Proc Natl Acad Sci U S A *95*, 5752-5756.

Katoh, K., Misawa, K., Kuma, K., and Miyata, T. (2002) MAFFT: a novel method for rapid multiple sequence alignment based on fast Fourier transform. Nucleic Acids Res *30*, 3059-3066.

Larkin, M.A., Blackshields, G., Brown, N.P., Chenna, R., McGettigan, P.A., McWilliam, H., Valentin, F., Wallace, I.M., Wilm, A., Lopez, R., et al. (2007). Clustal W and Clustal X version 2.0. Bioinformatics *23*, 2947-2948.

Letunic, I and Bork, P. (2007) Interactive Tree of Life (iTOL): An online tool for phylogenetic tree display and annotation. Bioinformatics *23*, 127-128.

Maiti, R., Van Domselaar, G.H., Zhang, H., and Wishart, D.S. (2004). SuperPose: a simple server for sophisticated structural superposition. Nucleic Acids Res *32*, W590-594.

Meisenzahl, A.C., Shapiro, L., and Jenal, U. (1997) Isolation and characterization of a xylose-dependent promoter from *Caulobacter crescentus*. J Bacteriol *179*, 592-600.

Miller, J.H., ed. (1972) Experiments in molecular genetics. Cold Spring Harbor Laboratory Press, Cold Spring Harbor, NY.

Möll, A. and Thanbichler, M. (2009) FtsN-like proteins are conserved components of the cell division machinery in proteobacteria. Mol Microbiol *72*, 1037-1053.

Murshudov, G.N., Vagin, A.A., and Dodson, E.J. (1997) Refinement of macromolecular structures by the maximum-likelihood method. Acta Crystallogr D Biol Crystallogr *53*, 240-255.

Potterton, L., McNicholas, S., Krissinel, E., Gruber, J., Cowtan, K., Emsley, P., Murshudov, G.N., Cohen, S., Perrakis, A., and Noble, M. (2004). Developments in the CCP4 molecular-graphics project. Acta Crystallogr D Biol Crystallogr *60*, 2288-2294.

Stamatakis, A., Hoover, P., and Rougemont, J. (2008) A rapid bootstrap algorithm for the RAxML web servers. Syst Biol *57*, 758-771.

Stock, D., Perisic, O., and Löwe, J. (2005) Robotic nanolitre protein crystallisation at the MRC Laboratory of Molecular Biology. Prog Biophys Mol Biol *88*, 311-327.

Thanbichler, M., Iniesta, A.A., and Shapiro, L. (2007) A comprehensive set of plasmids for vanillate- and xylose-inducible gene expression in *Caulobacter crescentus*. Nucleic Acids Res *35*, e137.

Thanbichler, M. and Shapiro, L. (2006) MipZ, a spatial regulator coordinating chromosome segregation with cell division in *Caulobacter*. Cell *126*, 147-162.

van den Ent, F., Lockhart, A., Kendrick-Jones, J., and Löwe, J. (1999) Crystal structure of the N-terminal domain of MukB: a protein involved in chromosome partitioning. Structure *7*, 1181-1187.
